# Spatial distribution of major and trace elements in artificial lakes in Serbia: health risk indices and suitability of water for drinking and irrigation purposes

**DOI:** 10.1007/s10661-023-11740-6

**Published:** 2023-09-21

**Authors:** Goran Marković, Aleksandar Ž. Kostić, Nebojša Đ. Pantelić, Radojka Maletić, Jana Štrbački, Jovan Cakić, Lazar Kaluđerović, Biljana P. Dojčinović, Angelo Maria Giuffrè, Jelena B. Popović-Djordjević

**Affiliations:** 1 Faculty of Agronomy Čačak, University of Kragujevac, Cara Dušana 25, 32000 Čačak, Serbia; 2 Faculty of Agriculture, University of Belgrade, Nemanjina 6, 11080 Belgrade, Serbia; 3 Faculty of Mining and Geology, Department of Hydrogeology, University of Belgrade, Djušina 7, 11 000 Belgrade, Serbia; 4 Faculty of Civil Engineering, University of Belgrade, Boulevard of the King Aleksandar 73, 11000 Belgrade, Serbia; 5 Institute of Chemistry, Technology and Metallurgy, National Institute of the Republic of Serbia, University of Belgrade, Njegoševa 12, Belgrade, Serbia; 6 Università degli Studi “Mediterranea” di Reggio Calabria Dipartimento di Agraria, Reggio Calabria, Italy

**Keywords:** Reservoirs, Potentially toxic elements, GIS, Health risk, Irrigation, Cluster analysis

## Abstract

Lakes and reservoirs are the main water resources for household, irrigation, and industrial use. Hence, it is necessary to control water pollution and have reliable information on water quality, which refers to its suitability for a particular purpose. The main objective of this study was to evaluate the suitability of water for drinking and irrigation purposes from ten artificial lakes on the territory of the Republic of Serbia. The physicochemical parameters were determined using standard analytical methods, whereas the optical emission spectrometry with inductively coupled plasma, ICP-OES, was used to determine the concentrations of 23 macro-, micro-, and trace elements in water. Health risk indices of drinking water and its suitability for irrigation purposes were evaluated. The results of physicochemical parameters, except for total organic matter, indicated that most samples were within the recommended values. Among analyzed elements, the concentration of Mg was elevated in the water of the lakes Srebrno and Prvonek. The results of health risk assessment clearly showed that there was no acute health risk (HQ_A_ < 1), whereas long-term risk (HQ_L_) in the studied lakes was observed with respect to As (lakes Grlište and Srebrno), Mn (lakes Srebrno and Prvonek), Ni (Lake Vrutci), and Sr (Lake Grlište). The irrigation criteria indicated that the water from the examined lakes had satisfactory quality for this purpose, except for residual sodium carbonates (RSC). This criterion indicated low quality or unsuitability of the water in 7 lakes. The main outcomes provided valuable information on the suitability of water from the artificial lakes for intended uses.

**Supplementary Information:**

The online version contains supplementary material available at 10.1007/s10661-023-11740-6.

## Introduction

Water covers the greatest part of Earth’s surface, and in its ocean and freshwater form, it represents a distinctive geographical feature of our planet. Water plays a fundamental role in all living beings. Furthermore, it bears great importance for the humankind from a cultural, political, and economic aspect. Since the ancient times, the management of water supply (consumption, sanitary use, agriculture and irrigation of land, fishery, navigation and transport, the building of hydropower plants…) and resources has been people’s primary concern. Such intense dependence of human societies on water underlines the importance of this natural resource for their existence (Hering, 2019). Although water scarcity is well recognized and its importance for life on Earth is indisputable, freshwater resources are often used non-adequately and they are unnecessarily polluted (Dobricic & Marjanovic, 2017). The resources of freshwater include groundwater, rivers, lakes, and reservoirs. These water bodies are useful or potentially useful to society regarding their various uses (Valencia-Avellan et al., 2017). Among them, inland lakes and artificial reservoirs represent important components of terrestrial hydrological systems and take part in the global water cycle. As dynamic and complex aquatic ecosystems, lakes are susceptible to climate change and human activity and may serve as indicators of climate and environmental changes (Li et al., 2020; Sheng et al., 2016).

Lakes and reservoirs are useful storages of freshwater with multiple purposes of use. They are available for domestic use, industrial use, agricultural production, energy supply, and recreational water use (Duan & Bastiaanssen, 2015). Sustainable agricultural production relies on water whose quality is satisfactory, meaning it causes no harm to crops that are intended for human (or animal) consumption (Dotaniya et al., 2022). However, the quality of surface water is a serious problem nowadays. Growing populations, anthropogenic impact, and natural processes have a negative effect on surface water and diminish its quality for drinking and other activities (Varol et al., 2012). In Europe, about 50% of water from surface sources (rivers and lakes) and approximately 25% of groundwater are of rather low ecological status or impoverished chemical status (Apollaro et al., 2019). Moreover, water shortage is the problem that has emerged because of excessive water consumption and climate change over the last century. Water consumption has increased fourfold, whereas the number of people who are affected by water scarcity increased by more than 40% (Kummu et al., 2016).

The importance of water for life on Earth is indisputable. Due to the fact that lakes and reservoirs are the main water resources for multiple purposes, it is necessary to control water pollution and have reliable information on water quality, which refers to its suitability for a particular purpose. Contamination of the aquatic environment with metal ions has received great concern because of their toxicity, the abundance and persistence in the environment, and subsequent accumulation in aquatic habitats. Toxic and potentially toxic elements may accumulate in aquatic flora and fauna and become part of human food chain, posing various health problems. In most countries, periodic monitoring and assessment of water quality has recently become an essential matter (Dotaniya et al., 2022; Li et al., 2020; Varol et al., 2012). The examination and monitoring of lakes and reservoirs are relatively simple to do individually, but they become more difficult with a larger number of lakes across huge areas. Lakes across a vast region are too numerous to be surveyed with field-based methods in regular periods (Sheng et al., 2016). In the Republic of Serbia, there are around 150 accumulations of different uses, where water supply, energy supply, and irrigation are the most common reasons for their formation (Milojković et al., 2018; Stanković, 2000). Thus, the water quality monitoring represents the most important measure, especially for the protection of water supply sources (Dobricic & Marjanovic, 2017; Dotaniya et al., 2022).

The main purpose of this study was to appraise the quality of the water from artificial lakes on the territory of the Republic of Serbia, by monitoring typical physicochemical parameters and concentrations of 23 macro-, micro-, and trace elements. Additionally, health risk indices and suitability of water for drinking and irrigation purposes were evaluated.

## Materials and methods

Due to concerns about the quality and safety of water used for water supply and agricultural purposes, the present study focused on the examination of artificial lakes in different regions of the Republic of Serbia that are used for multiple purposes. In that respect, studied parameters (physicochemical properties, health risk indices, and irrigation quality parameters) of water were evaluated using the appropriate analytical and statistical methods.

### Study area and lakes’ description

**Fig. 1:**
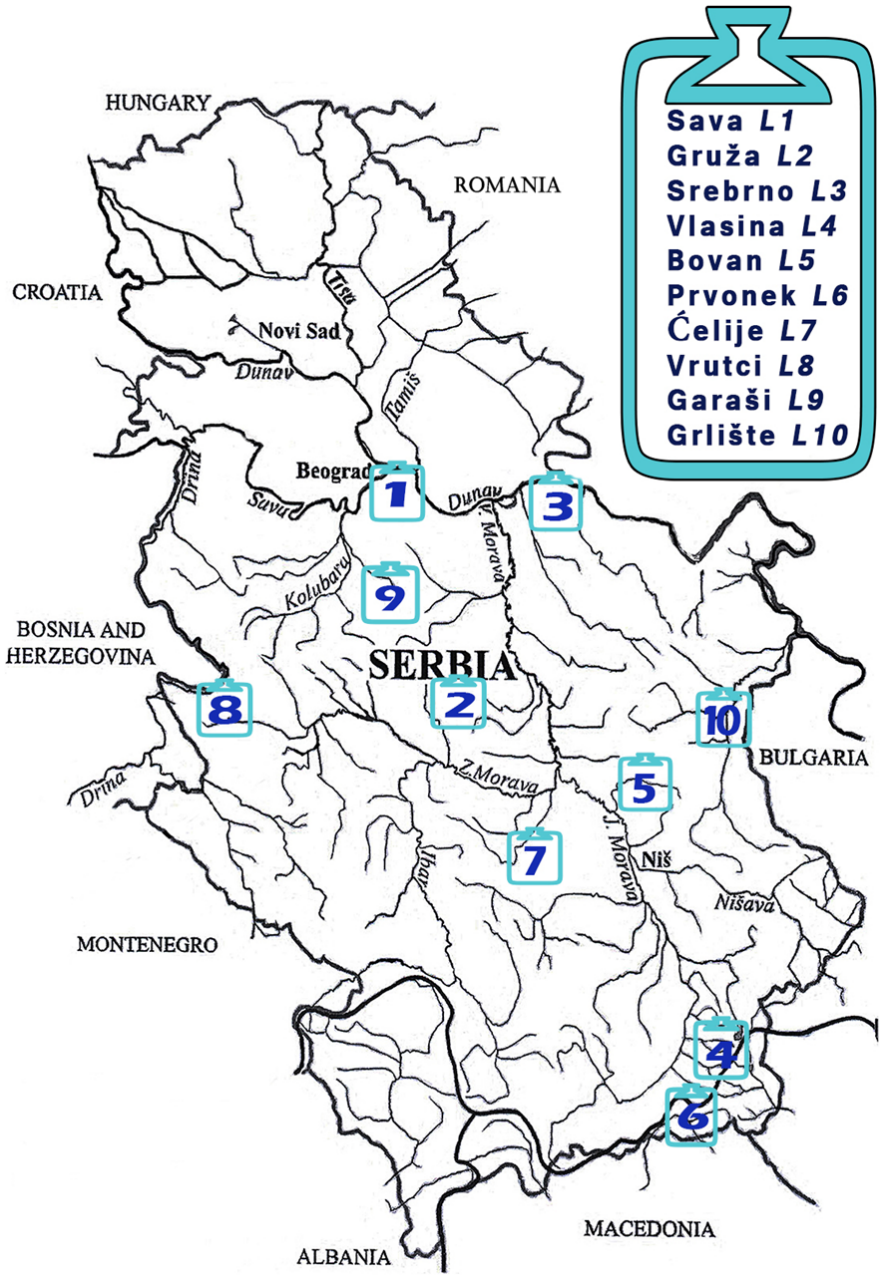
Geographical locations of the lakes within the study area

Ten artificial lakes (reservoirs) that were studied in this work are located within the territory of the Republic of Serbia that covers about 88,361 km^2^ (Milojković et al., 2018). Geographical locations and corresponding labels of the studied lakes are presented in Fig. 1. Coordinates and hydrological characteristics of the lakes are presented in Table S1 (Supporting information). Lakes Sava and Srebrno are formed as dammed arms of the two biggest rivers in Serbia, the Sava and the Danube, respectively. Vlasina Lake is the highest and largest semi-artificial lake in Serbia that has two permanent and about 30 floating islands, which represents one of the lake’s most famous features. Lakes Gruža, Bovan, Prvonek, Ćelije, Vrutci, Garaši, and Grlište were formed by damming the rivers with the intended use for water supply, irrigation, flood defense, and fishing (Dević et al., 2014; Kostić et al., 2016a; Martinović-Vitanović et al., 2009; Mićković et al., 2014; Milenković Andjelković et al., 2010; Stanković, 2000; Zlatković et al., 2010). There are relatively favorable conditions for the development of different biocenoses, primarily fish communities in the studied lakes. A high ichthyo production of the Cyprinidae family representatives was observed. The most common cyprinid species is *Carassius gibelio* (Simonović, 2006). In addition to cyprinids, the reservoirs are inhabited by large predators from other fish families—*Silurus glanis* (Siluridae), *Esox lucius* (Esocidae), and *Sander lucioperca* (Percidae) (Radenković, 2019), which is important for the maintenance of existing trophic networks as well as for recreational fishing.

### Geological background

*Lake Sava* (L1) is positioned in the lower middle Pleistocene sediment rocks. These rocks are dominantly limno-fluvial sediments: sand and gravel with *Corbicula fluminalis* fauna. Significant amounts of metal-bearing mineral were not detected (Marković et al., 1984).

*Lake Gruža* (L2) lies in Quaternary alluvial sediments surrounded by tertiary labradorite-andesites and pyroclastic labradorite-andesites. These andesites are more mafic than usual andesites. Monoclinic pyroxene is usually transformed into chlorite, carbonate, and secondary amphibole. Quaternary alluvial sediments are made of gravel, sand, and clay with different flora and fauna. Significant mineralization at the examined locality was not found, but some presence of coal was detected nearby in tertiary sediments as well as Pb–Zn minerals in dacite-andesite rocks near Kotlenik (Marković et al., 1963).

*Lake Srebrno* (L3) is situated in Quaternary alluvial and proluvial sediments surrounded by a piedmont diluvial-proluvial curtain. In the northwest part, near Ram fortress, low metamorphism crystalline schists occur. Alluvial sediments are mostly made of fine grade sediments (0.011–0.21 μm in diameter), with a high content of calcium carbonate (11.82–24.66%). These sediments are made of silty sand and sandy silt, with lenses made of fine gravel. From the mineralogical point of view, they contain a high content of mica (23.4%) and altered mineral grains (16.8–61.4%). Low metamorphism crystalline schists are metabasites and metasediments. Mineralogical analysis of the metasediments showed 52.64% of quartz, 23.63% of mica, 19.81% chlorite, 2.42% albite, and 2.42% apatite, titanite, and zircon (Rakić, 1978).

*Lake Vlasina* (L4) is placed on a series of metamorphic rocks surrounded by granitoids of Božica. These granitoids contain quartz, albite, and biotite with accessory minerals: apatite, zircon, and metallic minerals. Metamorphic series is presented by amphibole schists and green schists. In Paleozoic sediment-metamorphic ore deposits, iron is present in the form of magnetite in green schists. Magnetite is present in rocks abundant with chlorite. Magmatic intrusions during Alpine orogeny formed hydrothermal deposits of Pb, Zn, Cu, Mo, Sb, and Fe (Babović et al., 1977).

*Lake Bovan* (L5) is positioned in alluvial and terrace and Miocene sediments surrounded by the Riphean-Cambrian sericite and sericite-chlorite schists and Cambrian meta-sandstones. In schists, the most dominant minerals are sericite and quartz. The content of albite and chlorite varies. Accessory minerals are apatite, titanite, tourmaline, and opaque minerals. Miocene sediments lie overlap over crystal schists, Paleozoic and Mesozoic formations. At the examined locality, these sediments are part of the “red sediments” composed of conglomerates and coarse-grained sandstones. Significant mineralization was not detected at the examined locality, but the presence of bituminous coal and bituminous schists was detected in Aleksinac Miocene sediments (Krstić et al., 1974).

*Lake Prvonek* (L6) is located between biotite-muscovite schists on one side and gneisses on the other side. Biotite-muscovite schists are composed of quartz, muscovite, biotite, and variable content of albite, which determine the type of the rock. Biotite is often transformed into chlorite. Rocks are poor in accessory minerals. Garnets are the only minerals sometimes present in the form of coarse porphyroblasts. On the other hand, gneisses contain more accessory minerals such as apatite, metallic minerals, zircon, garnet, and titanite. Significant amounts of ore mineral were not detected at Lake Prvonek, but in the vicinity of the lake there are granitoids with Pb, Zn, Mo, Fe, Mn, and Cu minerals. Garnets contain Mn and they are relatively stable minerals. Mn^2+^ is a more migrative ion than Fe^2+^ and Fe^3+^. It precipitates as oxide or hydroxide. A higher oxidation potential is necessary for oxidation of Mn than it is for Fe. Neogene sandstones and conglomerates near Jovac village contain Mn in a sediment matrix in the form of pyrolusite (Babović et al., 1977).

*Lake Ćelije* (L7) is located in high-grade metamorphic biotite-muscovite-plagioclase schists. These schists are fine-grained rocks with main minerals: quartz, plagioclase, and micas (biotite in places transformed to chlorite). Garnets, kyanite, and staurolite are more frequent in these rocks than in gneisses. Other minerals that could be found are tourmaline, apatite, titanite, amphibole, epidote, magnetite, ilmenite, and pyrite. Mineral graphite was detected in these biotite-muscovite-plagioclase schists in the form of thin lenses (0.2–0.4 m thickness) (Rakić et al., 1969).

*Lake Vrutci* (L8) lies on the Dogger-Malm diabase-chert formation and ultramafic igneous rocks, lherzolites. The diabase-chert formation is separated on the lower and the upper part. The lithology of the upper part is very heterogeneous. Most abundant rocks are multicolored, thin-layered, and plate-shaped cherts, followed by massive sandstones with manganese coating, claystones, different limestone-dolomite rocks, and mafic igneous rocks. Lower parts of diabase-chert formation are made of diabase-chert formation sediments, changed by the contact of ultramafic intrusive, in situ. Most abundant are amphibolites, amphibolic schists, gabbro-amphibolites, actinolite-epidote schists, and epidotes. The lake is located near Zlatibor peridotite massive. In this massive, iron ore deposits, chromite, manganese magnesite, and dolomite occur (Mojsilović et al., 1971).

*Lake Garaši* (L9) is situated on granitic rock and different Paleozoic rocks like albite-chlorite-muscovite schists, sericite schists, sandstones, and quartzite. The metamorphic complex also contains sericite schists and phyllites. Granitic rocks represent the west part of the Bukulja complex: Šutica-Orlovac-Vagan zone (25 km). Petrochemical analysis indicated the presence of quartz (31%), plagioclase (34%), microcline (27%), biotite (5%), and muscovite (3%). Accessory minerals are zircon, apatite, and magnetite. At the Garaši Lake locality, the presence of sediments with metallic (Sn, Pb, Zn, and U) occurrences was detected. Tin (Sn) is present in mineral cassiterite in quartz veins, sometimes 1 m thick. In these veins, cassiterite, chalcopyrite, pyrite, and other Pb, Zn, and Cu minerals were also found. Near Garaši locality, U mineral (autunite) is present in disseminated concentrations in hydrothermally altered granitoids (Filipović et al., 1971).

*Lake Grlište* (L10) lies on alluvial sediments and Cretaceous rocks of different age. In the west, the Aptian sandstones, sandy limestones, and claystones, as well as Albian glauconitic sandstones and alevrolites were found. In the east, Cenomanian sandstones and claystones, as well as Turonian-Senonian marls and sandstones, were found. The examined locality is not rich in metallic occurrences. Bentonite clay was found north of Grlište and Fe was found in low concentrations in the form of limonite in glauconitic sandstones south of Grlište near Zagađe locality (Veselinović et al., 1967).

### Water sample collection

Samples were collected following the procedure described in our previous work during July and August 2017 (Kostić et al., 2016b). The water from the lakes was collected in sterile bottles from several sampling points within the lake. The individual samples from each lake, taken from 3 to 5 points at the depth of 30 cm, were mixed together in order to make a composite sample and filtered before further analyses. Additionally, water samples taken for multielemental analysis (ICP-OES) were placed in polypropylene bottles which were previously washed with HNO_3_ (1:1, v/v) and then thoroughly rinsed with ultrapure water. Each sample was stabilized by addition of 65% nitric acid (1 mL per 1 L of water). A total of 20 samples from 10 studied lakes were prepared, 10 for physicochemical parameters and 10 for multielemental analysis.

### Analyses of physicochemical parameters

Physicochemical parameters of the lake water samples were determined according to literature. The temperature (expressed in °C) of the samples was measured in situ just after sampling using a thermometer (Yu et al., 2009). pH value was measured with a pH meter (MM multimeter 41, pH electrode 50 21 T). Conductivity (expressed as μS/mL) was determined by a conductometer (Crison, Multimeter MM 41, EC cell 50 70). Alkalinity and acidity (expressed as mg/L CaCO_3_) were determined by titration of the water with a standard solution of sulfuric acid (0.05 mol/L), with methyl orange used as an indicator (Tsogas et al., 2010), and by titration with a standard NaOH solution (0.1 mol/L) in the presence of phenolphthalein as an indicator (Vogel, 1971), respectively. The total content of organic matter (expressed as mg/L) was determined by the Kubel-Tiemann method (titration with a potassium permanganate in acid solution) as recommended by the national regulations (Drinking Water, 1990). Chloride content in the water (expressed as mg/L) was determined by volumetric titration (More’s method) using a standard solution of silver nitrate (0.1 mol/L) with potassium chromate as an indicator (Waters-Doughty, 1924). Total hardness (TH) was calculated based on the concentrations of Ca and Mg, as reported in literature (Tsogas et al., 2010).

### Multielemental analysis

Analytical technique inductively coupled plasma-optical emission spectrometry (ICP-OES) was used for the determination of twenty-three elements: Ca, Mg, K, Na, Si (macroelements), Al, As, B, Ba, Co, Cr, Cu, Fe, Li, Mn, Ni, Pb, Sb, Se, Sr, V, and Zn (micro- and trace elements). The preparation of water samples and the measurement of the concentration of the elements were described in detail in our previous work (Kostić et al., 2016b). Briefly, water sample preparation was performed according to the standard US EPA Method 3015. Digestion was performed in a microwave digester model ETHOS 1 (Milestone, Italy). Digestion of the 45 mL sample was done with 5 mL of HNO_3_ (65%, Suprapur®, Merck, Darmstadt, Germany) in the following temperature program: 10 min at 160 °C and then another 10 min at 165 °C. Analytical technique, inductively coupled plasma optical emission spectrometry (ICP-OES) (iCAP 6500 Duo ICP, Thermo Fisher, Cambridge, UK) was used to quantify the elements in the sample solution. Standards for the instrument calibration were prepared based on multielemental certified reference solution ICP Standards: Silicon, Plasma Standard Solution, Specpure®, Si 1000 µg/mL and Multielement Plasma Standard Solution 4, Specpure® (Alfa Aesar GmbH and Co KG, Germany) and SS-Low Level Elements ICV Stock (10 mg/L) (VHG Labs, Inc. Part of LGC Standards, Manchester, NH 03103, USA). The certified reference material EPA Method 200.7 LPC Solution (ULTRA Scientific, USA) was used for analytical process quality control. The obtained recovery for all elements was in the range of 97–103%. The limit of detection (LOD) and limit of quantification (LOQ) are given in Table S2 (Supporting material). Results are presented as mean values of triplicate measurements and standard deviation.

### Geographic information system (GIS)

In addition to the analytical processing of the results themselves, a graphical analysis of the obtained results was done. For graphical analysis and presentation of the results, ESRI ArcMap 10.1 GIS software environment was used. A database was created where the entities were 10 sampling lakes, while the attributes of the values or concentrations of individual elements were at the sampling sites themselves. By interpolating these results using the Inverse Distance Weighting (IDW) method, values or concentrations of individual elements outside the sampling sites were obtained. Inverse Distance Weighting (IDW) is an interpolation method that estimates values outside of the sampling locations as the average attribute values of all locations in the environment, proportional to the distance between them. In most cases, this value is the weighted arithmetic mean of all known surrounding locations, where the coefficient (*P*) is the mutual distance to the sampling location (Singh & Verma, 2019). The concentrations of analyzed elements were expressed in mg/L (for macroelements) and in μg/L (for micro- and trace elements) at six intervals for each element individually.

### Health risk assessment

Based on the results obtained by ICP-OES analysis for element content, theoretical calculations for two parameters (acute health risk quotient (HQ_A_) and long-term health risk quotient (HQ_L_)) were performed according to procedures described in our previous research. Tolerable weekly intake (TWI) and referent dose oral (RfD_oral)_ values were used for calculations (Kostić et al., 2016b).

### Irrigation water quality indices

Several basic criteria for evaluating water quality for irrigation purposes were determined: salinity hazard, sodium hazard, residual sodium carbonates, and specific ion toxicity. Salinity hazard of irrigation water was assessed by measuring its electrical conductivity (EC, in μS/cm), while sodium hazard was expressed by sodium adsorption ratio (SAR), calculated based on the concentrations of Na^+^, Ca^2+^, and Mg^2+^ ions, expressed as meq/L (Richards, 1954):

$$SAR=\frac{{Na}^{+}}{\sqrt{\frac{1}{2}({Ca}^{2+}+{Mg}^{2+})}}$$

The Wilcox diagram, which combines salinity and sodium hazard criteria, was used for the classification of analyzed lake water (Wilcox, 1955). As indicated by Eaton (1950), residual sodium carbonates (RSC), a parameter that indicates the additional sodium hazard, associated with calcium carbonate (CaCO_3_) and magnesium carbonate (MgCO_3_) precipitation, was calculated using the following equation (all concentrations in meq/L):

$$RSC=\left({CO}_{3}^{2-}+{HCO}_{3}^{-}\right)-({Ca}^{2+}+{Mg}^{2+})$$

Specific ion toxicity, which affects sensitive crops, was assessed for chloride and boron ions in the examined lake water samples according to Ayers and Westcot (1985).

### Statistical analysis

Characterization and classification of investigated lakes water were conducted using hierarchical cluster analysis (HCA) (Güleret al., 2002). Factor analysis (FA) was used to identify the main hydrogeochemical processes that influence the chemical composition of the lakes’ water (Davis, 1986). All statistical calculations were carried out in IBM SPSS Statistics 19.0 software.

The hierarchical cluster analysis was carried out using WPGMA (weighted pair group method, with arithmetic mean) as described by (Sokal & Michener, 1958):

$$D\left(O_i,O_j\right)=\Sigma d_s/n_in_j$$

The Euclidean distance was used as a measure of similarity between the lake water samples:

$$d_{{ij}} = \sqrt {\sum\limits_{{k = 1}}^{p} {\left( {X_{{ik}} - X_{{jk}} } \right)^{2} } }$$

The Kruskal–Wallis test was subsequently applied to examine whether there are statistically significant differences (at *α* = 0.05) between the isolated clusters (Kruskal et al., 1952).

The factor analysis was conducted based on the principal component method, and the number of the extracted factors was determined based on Kaiser’s criterion (Kaiser, 1960). To facilitate the interpretation of the extracted factors, varimax orthogonal rotation was applied (Kaiser, 1958). The Anderson–Rubin method was used for the calculation of factor scores (Anderson & Rubin, 1956).

## Results and discussion

### Physicochemical parameters of the water in the studied lakes

**Fig. 2:**
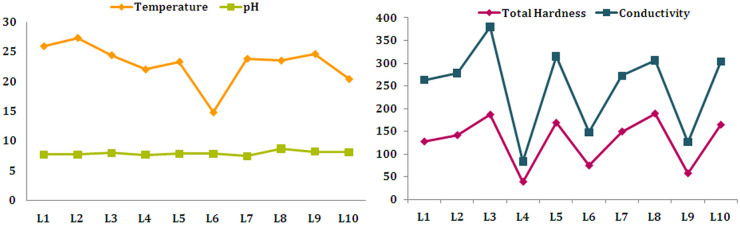
Temperature (°C), pH, total hardness (mg/L CaCO_3_), and conductivity (μS/mL) of the water in the studied lakes

**Fig. 3:**
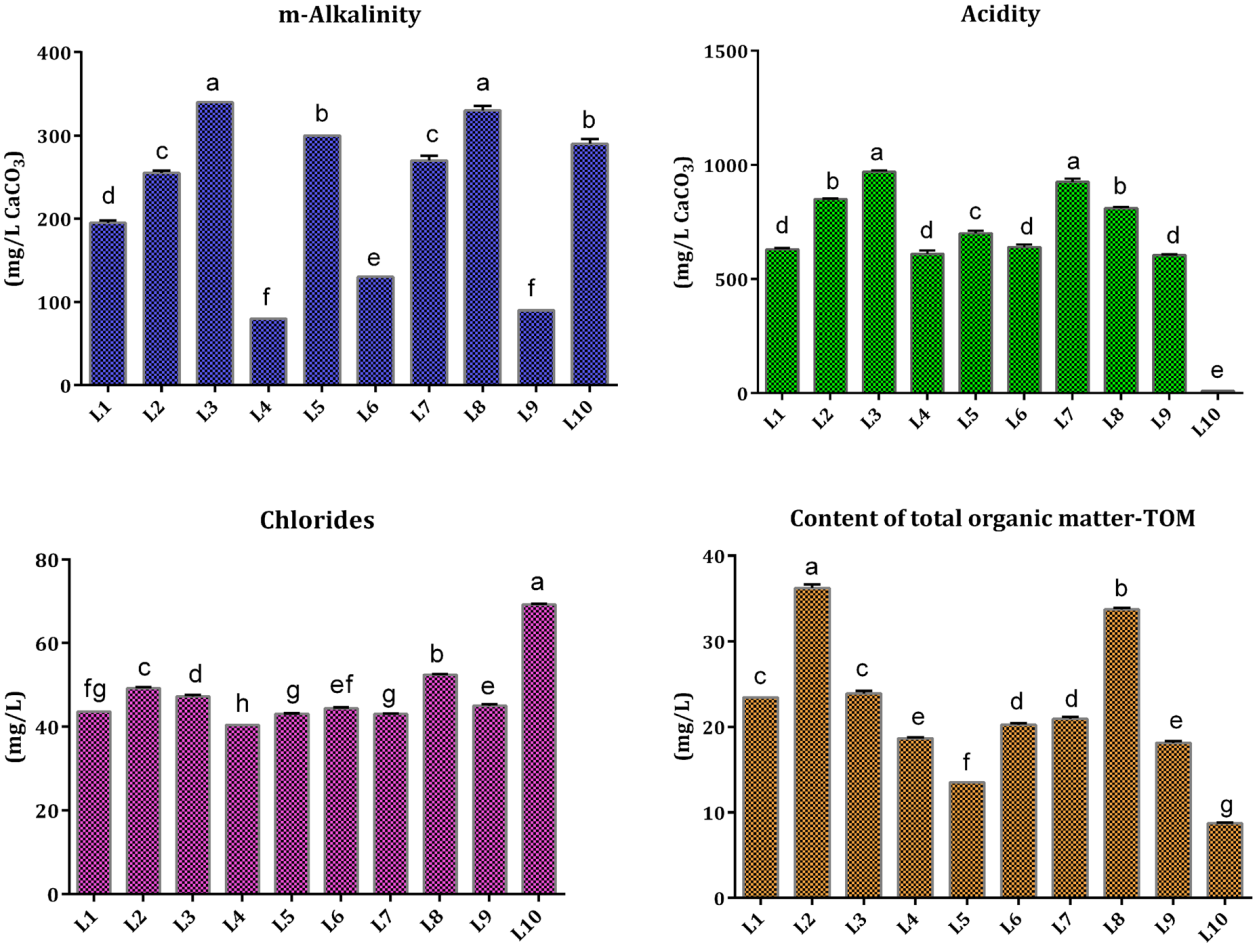
Results of selected physicochemical parameters of the water in the studied lakes. Values are presented as mean ± standard deviation. Different letters (a, b, c…) present statistically significant differences between the samples (*P* < 0.05)

Many chemical compounds that reach the environment from anthropogenic sources affect the quality of the aquatic ecosystem and water use for various purposes. Hence, for the purpose of controlling the release of such substances into the aquatic environment and water management, measures that are based on water quality criteria have been developed (UNECE, 1994). Knowing the basic physicochemical parameters of water may be helpful in making a rough assessment of the water quality category. Herein, the results of typical physicochemical parameters of the analyzed water samples are presented in Figs. 2 and 3.

Water temperature controls the rate of metabolic and reproductive activities and life cycles for the aquatic ecosystem (Carr & Neary, 2006). If the temperature is too high, metabolic activities can be accelerated or slowed, which can cause a complete cessation of the life cycle in water (Murdoch et al., 2001). Recorded temperatures of the tested samples ranged from 14.8 to 27.3 °C (Fig. 2).

pH value is one of the most easily measured water quality factors and can be influenced by various factors (Kostić et al., 2016b). The obtained results indicate that the investigated water samples were neutral to moderately alkaline. The lowest pH value (7.45) was recorded in Lake Ćelije (L7), while the highest value (8.67) was determined in Lake Vrutci (L8) (Fig. 2). The pH value of the water sample L8 was slightly above the limit value for drinking water proposed by national regulations (Official Gazette of RS, 2014) and EPA (2009). The results were in line with the data reported in literature (Dević et al., 2014).

Hardness (TH) of natural water originates mostly from dissolved calcium and magnesium salts. These two alkaline earth metals have an important role in fish’s metabolic processes, such as bone formation and blood clotting (Rahman et al., 2015). According to Boyd (2015), the total hardness of water for water supply can be classified as soft (< 50 mg/L), moderately hard (50–150 mg/L), hard (150–300 mg/L), and very hard (> 300 mg/L). The obtained results for TH (Fig. 2) indicate that the tested samples belong to soft (L4), moderately hard (L1, L2, L6, L7, L9), and hard (L3, L5, L8, L10) water.

Conductivity is the electrical property of water and depends on the amount of dissolved salts, the charge of the ion, and their mobility and it is considered to be a good indicator of quantitative mineral content in water (Pantelić et al., 2017). The obtained results for conductivity (Fig. 2), except in the sample L4, are comparable to those reported by Dević et al. (2014) which studied 28 Serbian lakes. Studies conducted on the lakes in surrounding countries such as Lake Ohrid, North Macedonia (Jordanoska et al., 2012), Lake Skadar, Montenegro (Pešić et al., 2018), and Lake Iyhie, Romania (Mihaiescu et al., 2012), support the results of the present study as well. On the other hand, even the lowest value of measured conductivity (83.5 μS/cm) in L4 was multiple times higher than that determined in some glacial lakes in Romania (Pop et al., 2013).

Alkalinity primarily originates from the content of carbonates, bicarbonates, and hydroxides in water. Moreover, concentrations of silicates, phosphates, and other base species could contribute to alkalinity (Chapman & Kimstach, 1996). Most tested samples had total alkalinity values above 200 mg/L (Fig. 3), and according to the classification of surface freshwater for the maintenance of aquatic life, they belong to the first-class category (UNECE, 1994). On the other hand, the analyzed samples from the lakes Vlasina (L4) and Garaši (L9) with the values of 80 and 90 mg/L, respectively, belong to the third class (UNECE, 1994). The results obtained for the samples L3, L5, and L8 are comparable to those determined in Lake Ohrid, North Macedonia (Jordanoska et al., 2012), while the values for the samples L4 and L9 are similar to those observed in Lake Blidinje, Bosnia and Herzegovina (Ivanković et al., 2011). It should be noted that the alkalinity value above 40 mg/L is considered to be an appropriate medium for good fish productivity (Sugunam, 1995).

Acidity of water is controlled by strong mineral acids, hydrolyzing metal salts, and weak acids such as carbonic, humic, and fulvic (Chapman & Kimstach, 1996). The acidified water releases potentially toxic dissolved metals from the sediments, which can adversely affect the aquatic ecosystem (Mosley et al., 2014). The results demonstrated that the highest acidity value was observed in Lake Srebrno (L3) which does not differ significantly from the value obtained for Lake Ćelije (L7) (*P* < 0.05). On the other hand, the lowest value of this parameter was recorded in Lake Grlište (L10) which was significantly different compared to other analyzed samples (Fig. 3).

Total organic matter (TOM) arises from the living material, and it is directly related to plant photosynthesis process, but it can also be associated with many waste materials in water and therefore TOM could be an indicator of pollution degree. According to literature (Chapman & Kimstach, 1996), concentrations of TOM in surface waters are normally lower than 10 mg/L, and the results of this study have shown that only the sample from Lake Grlište (L10) had a value below that, while the concentrations of this parameter for other tested samples ranged from 13.5 to 36.2 mg/L (Fig. 3). However, besides anthropogenic pollution, geology of the terrain may also cause the presence of organic matter.

Chlorides naturally occur in freshwater due to the dissolution of chloride-containing minerals. Additionally, it appears from industrial and sewage effluents as well as from salting the roads in the winter period (Kelly et al., 2008; Nagpal et al., 1981). High concentration of chloride in surface water can have a negative influence on water quality. It is reported that the elevated concentration of chlorides can harm aquatic organisms (Winter et al., 2011). According to the British Columbia water quality guidelines, the maximum recommended value for chloride in freshwater is 150 mg/L, the concentration at which the aquatic organisms are protected from chronic effects (Nagpal et al., 1981). In the analyzed samples, the range of chlorides was from 40.40 to 69.20 mg/L (Fig. 3). The obtained results were far below the maximum allowed chloride concentration for drinking water recommended by the World Health Organization (WHO) (Chapman & Kimstach, 1996).

### Content of macro-, micro-, and trace elements in the water of the studied lakes

**Fig. 4:**
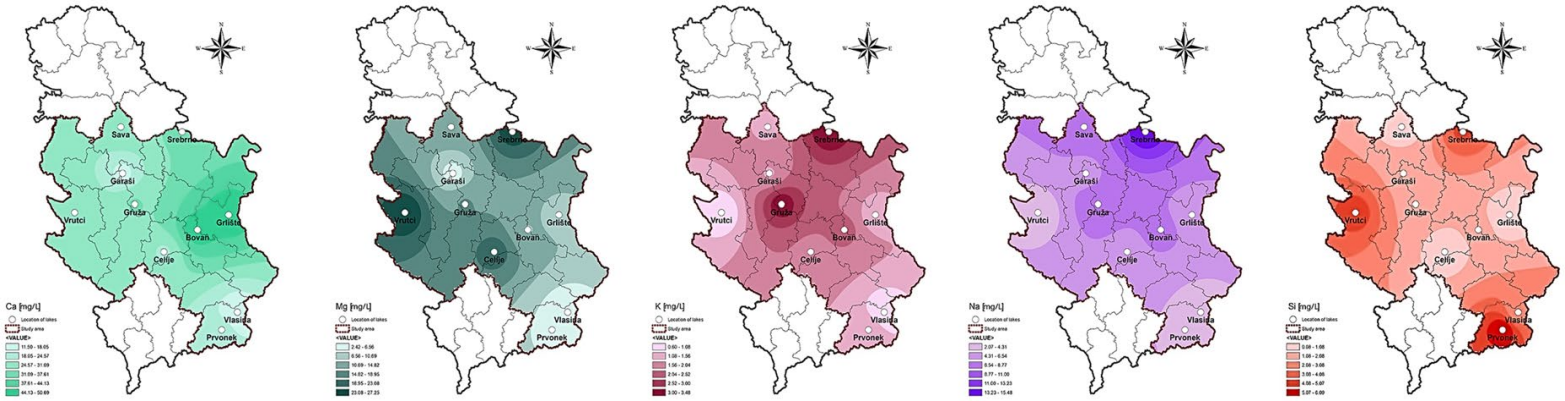
Distribution of macroelements (Ca, Mg, K, Na, and Si) in the water of the studied lakes

**Fig. 5:**
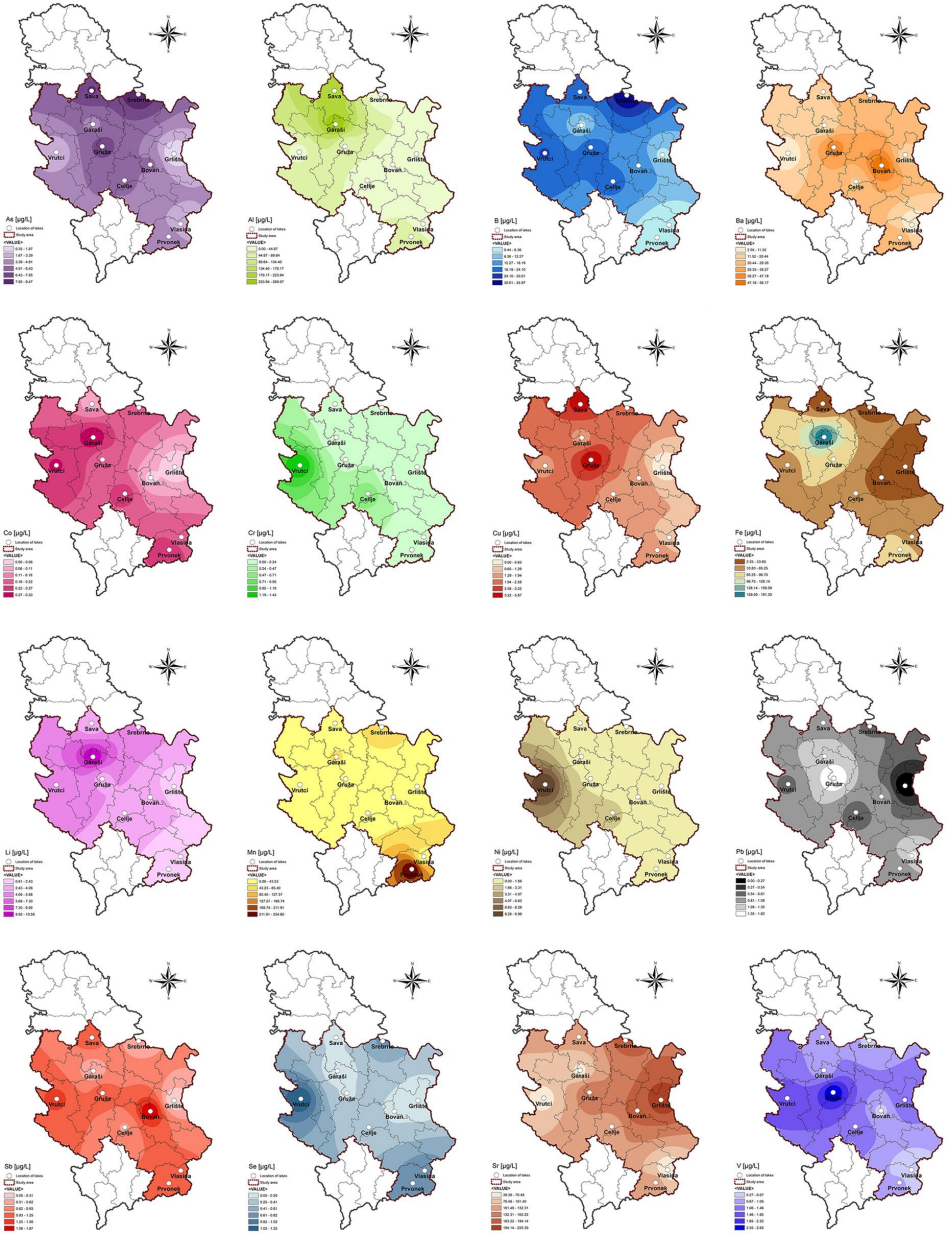
Distribution of micro- and trace elements (As, Al, B, Ba, Co, Cr, Cu, Fe, Li, Mn, Ni, Pb, Sb, Se, Sr, V) in the water of the studied lakes

Chemistry of water is affected by diverse factors, water–rock interactions as the dominant one as well as by other ecological and human aspects (Apollaro et al., 2019; Kostić et al., 2016b; Pantelić et al., 2019, 2020). Among 23 examined elements, concentrations of Zn in the water of all the studied lakes and Cd (with the exception in Lake Grlište) were below the limit of quantification (LOD). Results obtained for macro-, micro-, and trace elements are given in Tables S3 and S4 (Supporting material) whereas Arc-GIS was used to prepare geographic information system-based spatial distribution maps of the studied elements (Figs. 4 and 5). Concentrations of analyzed elements are expressed in mg/L (for macroelements) and in μg/L (for micro- and trace elements) at six intervals for each element individually, as shown in the maps’ legends.

*Macroelements*. Alkaline and alkaline earth metals (Li, Na, K, Mg, Ca, Sr, Ba) are major elements in water, whose presence is linked to natural processes (Kostić et al., 2016b). The most abundant elements in the water of the examined lakes were Ca, Mg, and Na with the highest concentrations recorded in lakes Glište (L10—50.69 mg/L), Vrutci (L8—27.25 mg/L), and Srebrno (L3—15.48 mg/L), respectively. Ca and Mg are the most abundant alkaline earth metals in the environment and major contributors to water hardness (Boyd, 2015). Concentrations of K and Si were below 10 mg/L in all studied water samples. Although silicon occurs in natural water (mostly in the form of silica acid), its concentration is rather low (2–25 and 0.5–3 mg/L in river and sea water, respectively) because of poor solubility of silicate minerals (Pantelić et al., 2019, 2020). Statistical analysis of macroelements content in the water revealed significant differences between most samples (*P* < 0.05). However, only the content of Mg significantly differed between all studied lakes (Table S3). Lake Vlasina had the lowest content of Ca, Mg, K, and Na. The highest content of K was measured in the lakes Gruža and Srebrno, whereas Lake Prvonek had the highest Si content. Results obtained for Si are in line with literature (Gršić et al., 2018; Pantelić et al., 2020). According to literature (Gršić et al., 2018), concentrations of four major elements in natural water are usually in the following order: Ca > Mg > Na > K, which was confirmed by the results of the present study. Dević et al. (2014) observed close association between lithology and water in 28 reservoirs in Serbia (Ca and HCO_3_ type of water). The obtained results for Ca, Mg, K, and Na in all water samples (L1–L10) were within WHO recommended values of water quality parameters for drinking water. Moreover, the results of the present study for Ca, Mg, and K were in good agreement with the findings of Dević et al. (2014). Distribution of macroelements based on the obtained results is depicted in Fig. 4.

*Micro- and trace elements*. Concentrations of micro- and trace elements were in the following ranges (min–max, in μg/L): Al (0.83–269.07), As (0.35–9.47), B (0.44–35.97), Ba (2.55–56.17), Co (0.04–0.33), Cr (0.02–1.43), Cu (0.96–3.87), Fe (2.25–191.30), Li (0.8–10.56), Mn (0.98–254.80), Ni (0.01–9.96), Pb (0.68–1.62), Sb (0.37–1.87), Se (0.15–1.2), Sr (57.44–225.30), and V (0.27–2.65). The lowest and highest concentrations were observed for Ni (0.01 μg/L; Lake Prvonek) and Al (269.07 μg/L; Lake Garaši), respectively. Cadmium (Cd) was detected only in Lake Grlište (0.04 μg/L) (Table S4).

For elements that are considered as primary contaminants (As, Ba, Cd, Cr(total), Cu, Hg, Pb, Sb, Se, Tl, and U), the Maximum Contaminant Levels (MCLs) in drinking water are set as shown in Table S5. MCL values are within the close range of Maximum Contaminant Level Goal (MCLG) that represents the level of the contaminant in drinking water below which there is no recognized or anticipated risk to health. MCLs are applicable norms (EPA, 2009). Several elements (As, Ba, Cd, Cr, Cu, Pb, Sb, and Se) that match this classification were analyzed in this study (Table S4). Exposure to these elements during a prolonged period can lead to negative health effects to humans, such as increased blood pressure and cholesterol, decreased blood sugar, kidney and skin disorders, stagnation in physical or mental development in children, circulatory problems, and increased risk of cancer (EPA, 2009).

Content of As varied significantly between the water samples of the studied lakes. The highest concentration (9.47 μg/L), found in the water sample of Lake Srebrno, was close to MCL value (0.01 mg/L) set by EPA (2009). Sources of As in water could be erosion of natural sediments or human activities such as agricultural production or industrial waste outflow. Due to its toxicity (especially As(III) ion), concentrations of As above MCL can be harmful (EPA, 2009; Fiket et al., 2007). Near Lake Srebrno, presence of As was not recorded in geological reports (Rakić, 1978). On the other hand, thermal power plant Kostolac in the west, or Moldova Nouă mine in the east, could be a potential source of As in the water of Lake Srebrno. Moldova Nouă mine is a porphyry Cu-Mo deposit, already mined in Roman times. Some minerals in this deposit include As sulfides and secondary As minerals (Ilinca, 2012). Moreover, As is part of minerals such as arsenides, sulfides, and arsenates. On the other hand, magma usually has low content of As, which can crystallize in its early stages, in the form of pyrite, arsenopyrite, and tetrahedrite. In hydrothermal fluids, As is associated with S, Fe, Pb, and Sb. Secondary minerals are Fe arsenate and Fe oxide-hydroxide. Rainwater during the wet season solubilizes oxidized arsenic or secondary minerals and disseminates them into the ecosystems through floods or storm water (Rodrıguez et al., 2004).

Alkaline earth metal Ba was detected in the lowest concentration in Lake Vlasina, whereas the highest content of this element was recorded in the lakes Gruža and Bovan (44.16 and 56.17 μg/L, respectively). These values were four to fivefold higher than those reported for the Ba content in Lake Baikal (Rahmi et al., 2008), but comparable with the results reported for spring water (Fiket et al., 2007) and river water (Kabata-Pendias & Szteke, 2015). Nonetheless, Ba content was much lower compared to MCL value (2 mg/L) (EPA, 2009), which might be expected as Ba presence was not reported in geological reports on these two localities (Krstić et al., 1974; Marković et al., 1963).

Maximum concentrations of Co, Cr, Cu, Li (with exception in Lake Garaši), Ni, Pb, Sb, Se, V, and Cd (detected only in Lake Grlište) in the water of all the studied lakes did not exceed 10 μg/L. In this respect, content of Cd, Cr, Sb, and Se was within MCL values (expressed in mg/L) set for each element (Cd—0.005; Cr(total)—0.1; Sb—0.006; Se—0.05). For the content of Cu and Pb action levels of 1.3 and 0.015 mg/L, respectively, are proposed by EPA (2009). In the lakes L1–L10, both elements were measured in concentrations below action levels. Moreover, concentrations of several elements in some lakes were below LOD: Cr (Sava, Garaši, and Grlište), Co, Cu, Pb, and Sb (Grlište), Ni (Vlasina and Grlište), and Se (Garaši and Grlište). Results of the present study for Li, Ni, Se, and V were comparable with the results presented for Lake Baikal, while the concentrations of As, Co, and Sb were much higher in lakes L1–L10 than in Lake Baikal (Rahmi et al., 2008).

Secondary contaminants that undergo “non-enforceable guidelines” (recommended but not required for systems to act in accordance with guidelines) are Al, Cu, Fe, Mn, and Zn. High level of these elements in drinking water may have an effect on the skin and teeth as well as on taste, odor, and color of water (EPA, 2009). Concentration of Al greatly and significantly varied among the water samples, from 0.83 μg/L in Lake Bovan to 269.07 μg/L in Lake Garaši. In Lake Sava, 225.90 μg/L of Al was measured (not significantly different from Lake Garaši, Table S4). In the water of two lakes (Srebrno and Grlište), concentration of Al was below LOD. According to literature, concentration of Al could be up to 1000 μg/L in river water. The solubility of Al varies at different pH, and the lowest is at pH > 6; hence, the obtained results were expected due to the fact that pH of the water samples L1–L10 was in the range 7.45 to 8.67 (Kabata-Pendias & Szteke, 2015). Secondary Maximum Contaminant Level for Al is 0.05–2 mg/L, which indicates that the obtained results are within the proposed range (EPA, 2009).

Concentration of Fe significantly differed in all the studied water samples; great variations were observed especially between lakes Garaši and Grlište where the highest and lowest contents were detected (191.30 and 2.25 μg/L, respectively). The obtained results were in line with literature where it was reported that the range of Fe concentration in river water is 11–739 μg/L (Kabata-Pendias & Szteke, 2015). In Lake Baikal, 0.002–6.3 μg/L of Fe was measured (Rahmi et al., 2008), which is multiple times lower than what was observed in the present study. Content of Fe in lakes L1–L10 was below the Secondary Maximum Contaminant Level—0.3 mg/L (EPA, 2009). According to the geological map, near Lake Garaši limonitic sandstones with glauconite were found. The amount of Fe in sandstones was not economically significant (Veselinović et al., 1967).

Mn is a broadly distributed component of ores and rocks. Its concentration greatly varied among the studied lakes; the highest content of Mn was found in Lake Prvonek (254.8 μg/L) while in Lake Sava its content was < 1 μg/L (Table S4). According to EPA (2009), Secondary Maximum Contaminant Level for Mn is 0.05 mg/L. The content of this element in lakes Prvonek and Srebrno exceeded the EPA guideline value, while in Lake Garaši it was close to the limit value. When the obtained results are compared to literature, it could be noticed that substantially lower values were reported for Lake Baikal (Rahmi et al., 2008). On the other hand, the results were comparable to the average content of Mn in river water (Kabata-Pendias & Szteke, 2015) and spring water (Fiket et al., 2007). Higher concentration of Mn in Lake Prvonek can be associated with the existence of granitoids with Pb, Zn, Mo, Fe, Mn, and Cu minerals (Babović et al., 1977). According to the geological map, near Lake Garaši only Sn and U were detected. Lake Garaši is situated on granitic rock which means that there could be some amount of Mn associated with garnets, only not significant enough to be mapped (Filipović et al., 1971). On the other hand, near Lake Srebrno, Mn presence was not recorded in geological reports (Rakić, 1978).

Content of another alkaline earth metal Sr was among the highest measured in all studied lakes. Water of Lake Grlište was the richest with Sr (225.3 μg/L) whereas the water of Lake Vlasina had the lowest content of this element (39.38 μg/L) (Table S4). Presence of Sr in water is associated with limestone deposits rich in celestite, but it could be found in Ca minerals as well. However, concentrations of Sr and Ca are not directly correlated. According to the latest guidelines set by Canadian authorities (Federal-Provincial-Territorial Committee on Drinking Water (CDW)) (Strontium in Drinking Water, 2018), a maximum acceptable concentration (MAC) for total Sr in drinking water is 7.0 mg/L. The obtained results were similar to those for spring water (Fiket et al., 2007), river water (Kabata-Pendias & Szteke, 2015), and Lake Baikal water (Rahmi et al., 2008), but much lower than MAC (Strontium in Drinking Water, 2018).

The highest/lowest content of B was found in lakes Srebrno (35.97 μg/L) and Vlasina (0.44 μg/L), respectively. Common B species in natural water are boric acid, B(OH)_3_, BO^2−^, and other borates. In water, B can react with soluble Al salts and form Al-B insoluble complexes, which reduce the content of B (Kabata-Pendias & Szteke, 2015; Kostić et al., 2016b). Reported ranges of B concentration in the river and spring water were 1.5–150 and 2.17–20.40 μg/L, respectively (Fiket et al., 2007; Kabata-Pendias & Szteke, 2015) which corroborates the results of the present study. On the other hand, in most of the studied lakes, content of B was higher compared to its content reported in Lake Baikal (Rahmi et al., 2008). Spatial distribution maps of micro- and trace elements are presented in Fig. 5.

### Health risk indices

Apart from microbial contamination of water, chemical contamination of drinking water is an equally significant problem that is associated with numerous water-linked health problems. In order to fill in the data about the chemical composition of lake water, health risk assessment was performed. With the obtained data given in Table 1, it is possible to state, with more accuracy, that the examined samples are either safe or not safe for human consumption or any other purposes.

**Table 1:** Acute and long-term health risk quotients for the water of lakes L1–L10 regarding toxic and potentially toxic elements

	**TWI**^**1**^	**RfD**_**oral**_^**2**^	**Sava (L1)**		**Gruža (L2)**		**Srebrno (L3)**		**Vlasina (L4)**		**Bovan (L5)**		**Prvonek (L6)**		**Ćelije (L7)**		**Vrutci (L8)**		**Garaši (L9)**		**Grlište (L10)**	
Element	mg/L	mg/kg/day	HQ_A_^3^	HQ_L_^4^	HQ_A_	HQ_L_	HQ_A_	HQ_L_	HQ_A_	HQ_L_	HQ_A_	HQ_L_	HQ_A_	HQ_L_	HQ_A_	HQ_L_	HQ_A_	HQ_L_	HQ_A_	HQ_L_	HQ_A_	HQ_L_
As	0.01	0.0003	0.11	6.8∙10^−2^	0.10	6.2∙10^−2^	0.13	8.0∙10^−2^	3.8∙10^−2^	2.3∙10^−2^	7.3∙10^−2^	4.4∙10^−2^	5.5∙10^−2^	3.3∙10^−2^	7.2∙10^−2^	4.3∙10^−2^	3.0∙10^−2^	1.8∙10^−2^	6.4∙10^−2^	3.8∙10^−2^	4.9∙10^−3^	3.0∙10^−3^
B	1.19	0.2	2.7∙10^−3^	3.0∙10^−4^	2.5∙10^−3^	3.0∙10^−4^	4.2∙10^−3^	5.0∙10^−4^	5.2∙10^−5^	6.0∙10^−6^	2.1∙10^−3^	2.0∙10^−4^	8.9∙10^−5^	1.0∙10^−5^	2.4∙10^−3^	3.0∙10^−4^	2.9∙10^−3^	3.0∙10^−4^	6.3∙10^−4^	7.0∙10^−5^	4.1∙10^−4^	4.0∙10^−5^
Ba	1.47	0.2	1.9∙10^−3^	3.0∙10^−4^	4.2∙10^−3^	6.0∙10^−4^	2.7∙10^−3^	4.0∙10^−4^	2.4∙10^−4^	3.0∙10^−5^	5.4∙10^−3^	7.0∙10^−4^	2.7∙10^−3^	4.0∙10^−4^	1.9∙10^−3^	2.0∙10^−4^	4.8∙10^−4^	6.0∙10^−5^	1.0∙10^−3^	1.0∙10^−4^	1.5∙10^−3^	2.0∙10^−4^
Mn	2.8	0.14	4.9∙10^−5^	2.0∙10^−5^	9.6∙10^−4^	3.0∙10^−4^	3.5∙10^−3^	1.3∙10^−3^	8.8∙10^−4^	3.0∙10^−4^	4.8∙10^−4^	2.0∙10^−4^	1.3∙10^−2^	4.6∙10^−3^	5.7∙10^−4^	2.0∙10^−4^	2.0∙10^−4^	7.0∙10^−5^	2.4∙10^−3^	9.0∙10^−4^	1.0∙10^−4^	4.0∙10^−5^
Ni	0.084	0.02	2.5∙10^−4^	2.0∙10^−5^	2.3∙10^−3^	2.0∙10^−4^	5.4∙10^−4^	4.0∙10^−5^	0	0	3.3∙10^−4^	3.0∙10^−5^	1.7∙10^−5^	1.0∙10^−6^	3.4∙10^−3^	3.0∙10^−4^	1.7∙10^−2^	1.3∙10^−3^	8.9∙10^−4^	7.0∙10^−5^	0	0
Se	0.24	0.005	1.0∙10^−4^	9.0∙10^−5^	1.1∙10^−4^	9.0∙10^−5^	3.8∙10^−4^	3.0∙10^−4^	4.3∙10^−4^	4.0∙10^−4^	8.8∙10^−5^	8.0∙10^−5^	4.3∙10^−4^	4.0∙10^−4^	1.9∙10^−4^	2.0∙10^−4^	7.2∙10^−4^	6.0∙10^−4^	0	0	0	0
Sr	0.91	0.6	1.6∙10^−2^	4.0∙10^−4^	2.5∙10^−2^	7.0∙10^−4^	2.8∙10^−2^	8.0∙10^−4^	6.1∙10^−3^	2.0∙10^−4^	2.8∙10^−2^	8.0∙10^−4^	1.8∙10^−2^	5.0∙10^−4^	1.8∙10^−2^	5.0∙10^−4^	8.9∙10^−3^	2.0∙10^−4^	9.5∙10^−3^	3.0∙10^−4^	3.5∙10^−2^	1.0∙10^−3^

^1^*TWI* tolerable weekly element intake values (mg/L) used for HQ_A_ calculation (WHO, 2010, 2017)

^2^*RfD*_*oral*_ referent oral dose values (mg/kg/day) used for HQ_L_ calculation (US EPA, 2018)

^3^*HQ*_*A*_ acute health risk quotient

^4^*HQ*_*L*_ long-term health risk quotient

In the case of acute health risk assessment, based on HQ_A_ values, if this parameter has a value equal or higher than 1, it is considered as high/significant (Leung et al., 2008). The obtained results (Table 1) clearly show that there is no acute health risk for anyone who uses the water from these lakes, since all of the values were significantly lower than 1. The highest value (around 0.1) was obtained for As from samples L1, L2, and L3 (lakes Sava, Gruža, and Srebrno, respectively). However, they were ten times lower compared to the limit value.

Besides the possibility to cause some acute disorders, there are also risks for consumers to develop some chronic or even carcinogenic diseases if they use water with elevated element content. Sometimes it is possible that there are no acute disorders but there is a long-term risk. This is why it is important to determine long-term quotients (HQ_L_) along with HQ_A_. According to literature’s recommendation, any HQ_L_ quotient equal/higher than 10^−3^ should be considered as “not-negligible” (Momot & Synzynys, 2005). Based on the obtained results (Table 1), it can be observed that, unlike the acute risk, there are low but measurable long-term risks in water all of the studied lakes. Again, it was mostly triggered by the presence of As ranging from 3∙10^−3^ (Lake Grlište) to 8.0∙10^−2^ (Lake Srebrno). It means that there is a potential risk of developing some chronic/carcinogenic diseases for 3/1000 (Lake Grlište) and 80/1000 (Lake Srebrno) of water users. However, it should be pointed out that this is probably caused by a quite low RfD_oral_ value for As (0.0003 mg/kg of body weight) which is recently established by US EPA (2018). Namely, this value is 5000 times lower compared to the one that was previously used (1.5 mg/kg body weight) and recommended by the same agency before 2018. In addition, low but calculable risks (> 10^−3^) were noticed for Mn in lakes Srebrno (1.3∙10^−3^) and Prvonek (4.6∙10^−3^), as well as for Ni (1.3∙10^−3^) and Sr (1∙10^−3^) in lakes Vrutci and Grlište, respectively. Although these results present only theoretical calculations, monitoring must be performed for a longer period and then followed up by additional examination for confirmation.

### Assessment of water quality for irrigation

**Fig. 6:**
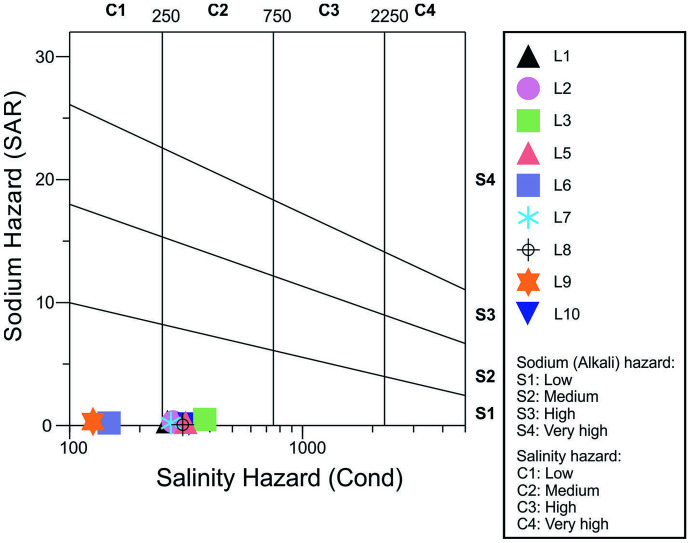
Wilcox diagram showing classification of 10 lake water samples, based on sodium and salinity hazard

Given the significant distribution of agricultural land in the vicinity of the examined lakes, the use of lake water for irrigation is one of its most important purposes. Inadequate quality of irrigation water has a direct negative impact on both soil and crops, so the examination and evaluation of the chemical composition of water are mandatory in the process of designing irrigation systems. Based on the physical and chemical parameters of the analyzed lake water samples, as well as their macro- and microelement content, the described irrigation criteria were calculated, and the results are presented in Table 2 and Fig. 6.

**Table 2:** Irrigation quality parameters for 10 lake water samples, recommended guideline values, and potential effects on crops

**Parameter**	**Sava (L1)**	**Gruža (L2)**	**Srebrno (L3)**	**Vlasina (L4)**	**Bovan (L5)**	**Prvonek (L6)**	**Ćelije (L7)**	**Vrutci (L8)**	**Garaši (L9)**	**Grlište (L10)**	**Referent values/potential effects on crops**
Conductivity (μS/cm)	263	278	380	83.5	315	147.7	272	306	125.9	303	< 750—no hazard 750–1500—some hazard 1500–3000—moderate hazard 3000–7500—severe hazard (Bauder et al., 2011)
SAR	0.303	0.308	0.493	0.171	0.251	0.201	0.202	0.066	0.32	0.167	0–10—low sodium hazard 10–18—medium sodium hazard 18–26—high sodium hazard > 26—very high sodium hazard (Wilcox, 1955)
RSC (mg/L)	1.36	2.28	3.08	0.83	2.63	1.12	2.42	2.83	0.66	2.52	< 1.25—safe 1.25–2.5—marginal > 2.5—unsuitable (Eaton, 1950)
Cl (meq/L)	1.23	1.39	1.33	1.14	1.21	1.25	1.21	1.48	1.27	1.95	< 2—generally safe for all plants 2–4—sensitive plants usually show slight to moderate injury 4–10—moderately tolerant plants usually show slight to substantial injury > 10—can cause severe problems (Bauder et al., 2011)
B (mg/L)	0.023	0.021	0.036	0	0.018	0.001	0.02	0.025	0.005	0.004	< 0.5—satisfactory for all crops 0.5–1.0—satisfactory for most crops 1.0–2.0—satisfactory for semi-tolerant crops 2.0–4.0—satisfactory for tolerant crops only (Bauder et al., 2011)

According to the majority of criteria that have been considered in Table 2, the analyzed lake water samples are of satisfactory quality for irrigation. There is no salinity hazard, since the values of the electrical conductivity in all 10 water samples are below 750 μS/cm. Sodium hazard is also low (SAR < 10), and chloride and boron levels are acceptable (< 2 meq/L and < 0.5 mg/L, respectively). The only parameter that indicates a potential water quality problem is RSC, since the proposed criterion is met only for lakes Vlasina, Prvonek, and Garaši, while the water from lakes Srebrno, Bovan, Vrutci, and Grlište is characterized as unsuitable for irrigation. This is due to a relatively high alkalinity of this water, which makes it susceptible to calcium carbonate and magnesium carbonate precipitation, when the soil solution becomes concentrated through evapotranspiration (Zaman et al., 2018). This eventually causes the SAR value to rise above the initially calculated values and the sodium hazard increases. To amend these high RSC values, the addition of calcium-containing amendments, such as gypsum (CaSO_4_ × 2H_2_O), is recommended (Shahid et al., 2018).

Based on the Wilcox diagram, Fig. 6, most of the lake water samples were classified as C2-S1, i.e., medium salinity hazard and low sodium hazard, and the samples L6 and L9 were classified as C1-S1, i.e., low salinity hazard and low sodium hazard. This minor classification discrepancy, in relation to Table 2, is a consequence of different guideline values for electrical conductivity (Wilcox, 1955). Nevertheless, all analyzed lake water samples are suitable for irrigation of plants with moderate salt tolerance and on most soils, without any special practices for salinity control (Wilcox, 1955). Due to the lowest electrical conductivity value (83.5 μS/cm) in sample L4 (Lake Vlasina), it was not shown in the diagram, since the conductivity axes start at 100 μS/cm.

### Multivariate statistical analysis

#### Hierarchical cluster analysis (HCA)

**Fig. 7:**
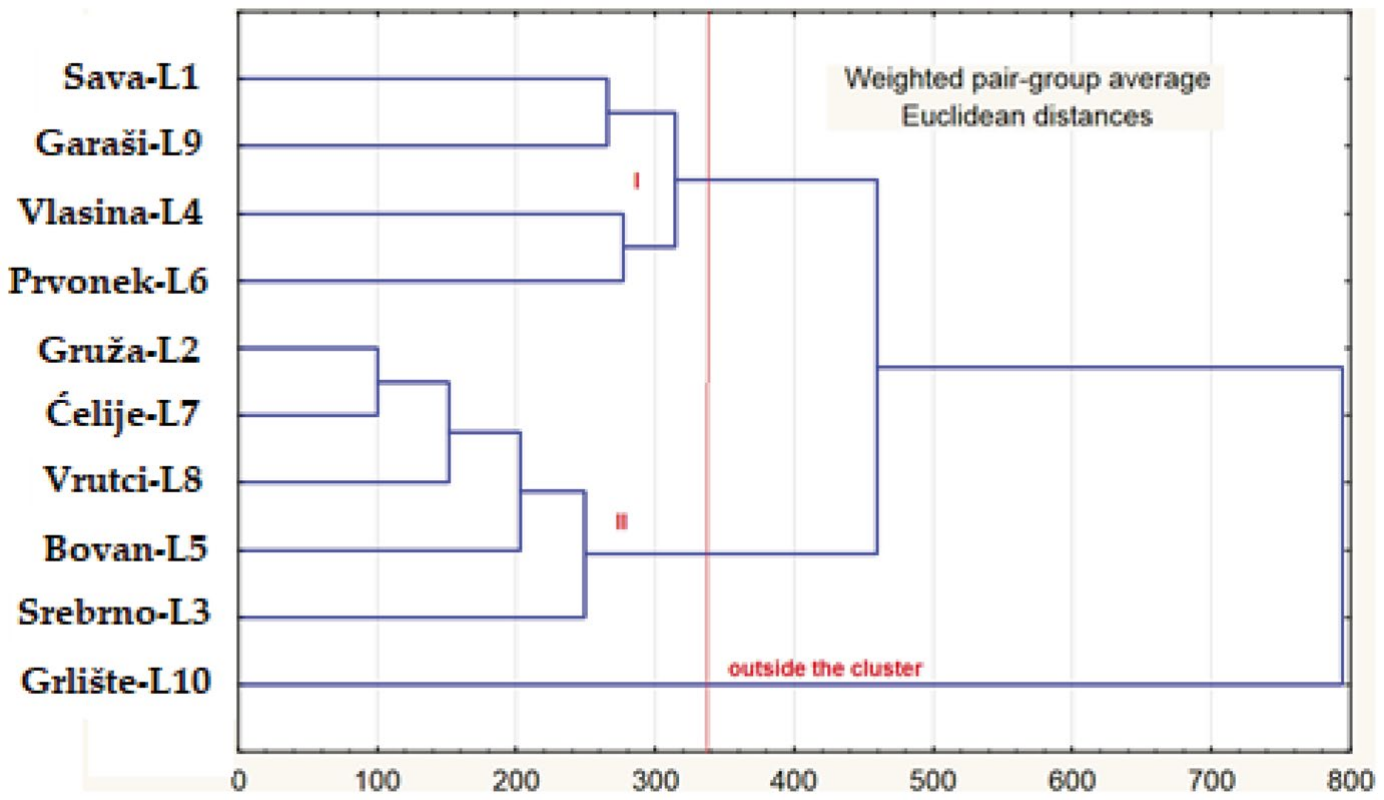
Dendrogram of water samples from ten lakes (WPGMA, Euclidean distance)

Hierarchical cluster analysis was conducted based on the reported concentrations of macro-, micro-, and trace elements (mg/L and μg/L, respectively), of 10 lake water samples (Tables S3 and S4). The results are shown in a dendrogram (Fig. 7). Two homogeneous groups of lakes are distinguished, while sample L10 (from Lake Grlište) remained outside the clusters, due to its several specific hydrochemical features. The subsequent Kruskal–Wallis test confirmed that there are statistically significant differences (at *α* = 0.05) between these two clusters.

**Fig. 8:**
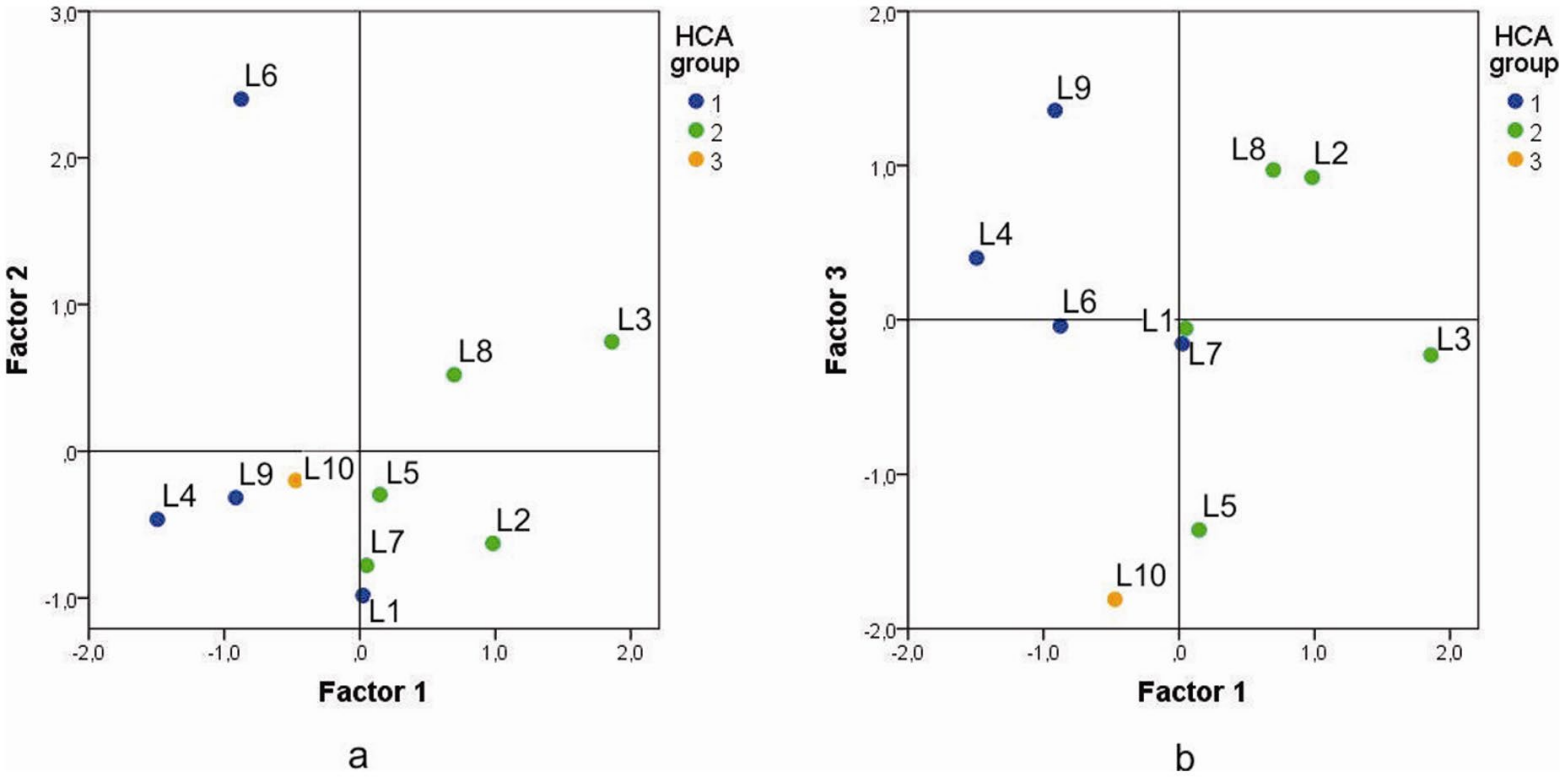
Plots of factor scores of 10 lake water samples, labeled according to HCA groups. Factor 1 vs. factor 2 (**a**) and factor 1 vs. factor 3 (**b**)

The first cluster consists of four lakes: Sava, Garaši, Vlasina, and Prvonek. It is characterized by low electrical conductivity (average value, 155.0 μS/cm), as well as low concentrations of macroelements (average values, Ca—19.4 mg/L, Mg—6.3 mg/L, and Na—5.0 mg/L). Compared to the rest of the analyzed lake water samples, this cluster features elevated concentrations of Fe (83.7 μg/L) and Mn (80.1 μg/L). Cluster 2 comprises the following lakes: Gruža, Ćelije, Vrutci, Bovan, and Srebrno. The average electrical conductivity of these water samples is 310.2 μS/cm, which is twice as much as that in cluster 1, and the recorded water temperatures are also higher (23.3–27.3 °C). Concentrations of macroelements were greater compared to the first cluster (average values, Ca—33.7 mg/L, Mg—20.1 mg/L, and Na—7.8 mg/L). On the other hand, Fe and Mn average concentrations were notably lower than in cluster 1 (39.5 and 22.7 μg/L, respectively). Lake Grlište (outside the clusters, Fig. 8) was characterized by the lowest recorded TOM value (8.7 mg/L), but also the highest Ca concentration (50.7 mg/L). It showed the lowest concentrations of Fe (2.25 μg/L) and As (0.35 μg/L) and overall low concentrations of analyzed macro- and microelements.

#### Factor analysis (FA)

Factor analysis was performed on 11 selected parameters: total organic matter (TOM, mg/L), conductivity (EC, μS/cm), pH, temperature (°C), Ca (mg/L), Mg (mg/L), Na (mg/L), K (mg/L), Si (mg/L), Fe (μg/L), and Mn (μg/L), measured in 10 lake water samples. The reduction of the total number of analyzed parameters was necessary in order to obtain an appropriate dataset for this type of analysis. Three factors were extracted, which explained the total of 71.7% variance of the analyzed data. These three factors, their factor loadings (only significant loadings are shown, absolute value > 0.4) and the amount of variance explained, are given in Table 3.

**Table 3:** Factor loadings and explained variance (in %) of three extracted factors, with varimax

**Parameter**		**Factor**	
	1	2	3
Conductivity	0.869	/	− 0.428
Mg	0.846		
Na	0.736		
K	0.722		
Si		0.900	
Mn		0.887	
Temperature	0.507	− 0.778	
pH			
Ca	0.418		− 0.764
Fe			0.713
TOM	0.545		0.707
Explained variance (%)	31.5	21.4	18.8
Cumulative variance (%)	31.5	52.9	71.7

Factor 1 was characterized by medium to high positive factor loadings for electrical conductivity, temperature, and TOM, as well as macroelements Mg, Na, K, and Ca. This was the dominant factor for the analyzed dataset, since it explains the largest amount of the variance (31.5%). Factor 2 explained 21.4% of the variance and comprises high positive loading for Si and Mn, together with high negative loading for temperature. Factor 3 explained a slightly lower amount of variance (18.8%) compared to factor 2 and it consisted of high positive factor loadings for Fe and TOC, together with high negative factor loading for Ca and moderately high negative factor loading for electrical conductivity.

Factor scores were calculated in order to facilitate the interpretation of the extracted factors and to connect them with individual lake water samples. The factor score plots are given in Fig. 8, and the samples’ symbols indicate their HCA group membership.

It is apparent from Fig. 8 that all lake water samples from cluster 2 were primarily under the influence of factor 1. Namely, warmer water with higher electrical conductivity also has higher content of macroelements (Ca, Mg, Na, and K) and generally larger amounts of organic matter. Factor 2 indicated that the higher concentrations of Mn in the water were probably related to the dissolution of Mn silicates present in the area (such as garnets, at the locality of Lake Prvonek) and that this process is more intense in colder water. This was the case with the water from Lake Prvonek (sample L6, Fig. 8), where maximal Mn and Si contents in this study were recorded (254.80 μg/L and 6.09 mg/L, respectively), as well as minimal temperature (14.8 °C). Besides garnets, contained in the biotite-muscovite schists, the probable source of manganese is pyrolusite, present in Neogene sandstones and conglomerates, at Lake Prvonek locality. Lake Srebrno (sample L3, Fig. 8) also has elevated manganese content (69.30 μg/L) and moderately elevated Si content (4.36 mg/L), while the recorded water temperature (24.4 °C) was substantially higher than in Lake Prvonek. Factor 3 pointed out the connection between higher Fe content and the presence of organic matter in lake water, followed by low Ca concentrations. This particularly refers to Lake Garaši (sample L9, Fig. 8), with pronounced Fe content (191.30 μg/L), elevated TOM (18.10 mg/L), and low Ca concentration (15.49 mg/L). Biotite, magnetite, chalcopyrite, pyrite, and other Fe minerals were detected in the Lake Garaši area (Filipović et al., 1971), and these are the presumed sources of Fe in water.

## Conclusions

The results of the study provided insight into physicochemical characteristics and spatial distribution of elements in 10 artificial lakes in Serbia which are mainly intended for water and energy supplies, irrigation, and recreational purposes. Typical physicochemical parameters in the water of most of the studied lakes were within recommended values; exceptions were observed for pH in Lake Vrutci (slightly above the limit value for drinking water proposed by national regulations and EPA) and total organic matter (TOM) content that was elevated in all the samples except in Lake Grlište. Among major elements, Ca, Mg, and Na were the most abundant ones, whereas for micro- and trace elements Si was measured in the highest concentration in the water of all the examined lakes. Zn and Cd (except for the water from Lake Grlište) were below the limit of quantification in all the water samples. Most toxic and potentially toxic elements that are considered as drinking water contaminants were within safe limit values. Only the content of Mn was above the EPA guideline value in lakes Prvonek and Srebrno. Health risk indices calculated for As, B, Ba, Mn, Ni, Se, and Sr indicated no significant acute risk, but long-term risk was observed for As and Sr (Lake Grlište), As and Mn (Lake Srebrno), Mn (Lake Prvonek), and Ni (Lake Vrutci). The irrigation criteria indicated satisfactory quality of water if used for this purpose, with the exception of residual sodium carbonates. Based on this criterion, water in lakes Sava, Gruža, and Ćelije was characterized as low quality or unsuitable for irrigation in lakes Srebrno, Bovan, Vrutci, and Grlište. However, continuous monitoring of water quality criteria in the examined lakes is recommended.

### Supplementary Information

Below is the link to the electronic supplementary material.

Supplementary file1 (DOC 209 KB)

## Data Availability

Data is contained within the article.

## Declarations

### Disclaimer

All authors have read, understood, and complied as applicable with the statement on “Ethical responsibilities of Authors” as found in the Instructions for Authors and are aware that with minor exceptions, no changes can be made to authorship once the paper is submitted.
