# Supplementary material for: Spatial distribution of major and trace elements in artificial lakes in Serbia: health risk indices and suitability of water for drinking and irrigation purposes
Source: Environ Monit Assess. 2023 Sep 21;195(10):1237. doi: 10.1007/s10661-023-11740-6 (PMC10514149; doi:10.1007/s10661-023-11740-6)
Supplement: Supplementary file 1 — Supplementary file1 (DOC 209 KB) [file 10661_2023_11740_MOESM1_ESM.doc]

**Spatial distribution of major and trace elements in artificial lakes in Serbia: health risk indices and suitability of water for drinking and irrigation purposes**

Goran Marković^1^, Aleksandar Ž. Kostić^2^, Nebojša Đ. Pantelić^2^, Radojka Maletić^2^, Jana Štrbački^3^, Jovan Cakić^4^, Lazar Kaluđerović^1^, Biljana P. Dojčinović^5^, Angelo Maria Giuffrè^6*^, Jelena B. Popović-Djordjević^2*^

^1^ University of Kragujevac, Faculty of Agronomy Čačak, Cara Dušana 25, 32000 Čačak, Serbia

^2^ University of Belgrade, Faculty of Agriculture, Nemanjina 6, 11080 Belgrade, Serbia

^3^ University of Belgrade - Faculty of Mining and Geology, Department of Hydrogeology, Djušina 7, 11 000 Belgrade, Serbia

^4^ University of Belgrade, Faculty of Civil Engineering, Boulevard of the King Aleksandar 73, 11000 Belgrade, Serbia

^5^ University of Belgrade, Institute of Chemistry, Technology and Metallurgy, National institute of the Republic of Serbia, Njegoševa 12, Belgrade, Serbia

^6^ Università degli Studi "Mediterranea" di Reggio Calabria Dipartimento di Agraria, Reggio Calabria, Italia

***** Correspondence: [jelenadj@agrif.bg.ac.rs](mailto:jelenadj@agrif.bg.ac.rs)

[amgiuffre@unirc.it](mailto:amgiuffre@unirc.it)

| **Table of Contents** |
| --- |
| **Table S1.** Coordinates and hydrological characteristics of the lakes |
| **Table S2.** ICP-OES: Limit of detection (LOD) and Limit of quantification (LOQ) |
| **Table S3.** Concentrations of macro elements in water of studied lakes (mg/L) |
| **Table S4.** Concentrations of micro- and trace elements in water of studied lakes (μg/L) |
| **Table S5**. Limit values of national and international regulations and range of elements concentrations in the lake water samples L1-L10 |

**Table S1.** Coordinates and hydrological characteristics of the lakes

| **Sample** | **Lake** | **Coordinates /region** | **Elevation^1^ (m a.s.l.)** | **Depth**  **(m)** | **Watercourse** | **Intended use^2^** | **Type** | **Reference** |
| --- | --- | --- | --- | --- | --- | --- | --- | --- |
| **L1** | Sava | 44°49' N / 20°27' E  Central Serbia | 73 | 4.5 | Sava river | Recreation,  water supply | Artificial  (dammed arm of Sava) | Mićković et al., 2014 |
| **L2** | Gruža | 43°56' N / 20°41' E  Central Serbia | 267 | 27 | Gruža river | Water supply | Artificial | Dević et al., 2014 |
| **L3** | Srebrno | 44°46' N / 21°28' E  East Serbia | 70 | 8 | Danube river | Recreation | Artificial  (dammed arm of Danube) | Martinović-Vitanović et al., 2009 |
| **L4** | Vlasina | 42°42' N / 22°20' E  South Serbia | 1213 | 22 | Vlasina river | Water supply,  energy supply, recreation | Semi-artificial | Dević et al., 2014; Stanković, 2000 |
| **L5** | Bovan | 43°39' N / 21°43' E  South Serbia | 262 | 50 | Moravica River | Water supply,  flood defense, irrigation, industrial reservoir | Artificial | Dević et al., 2014; Stanković, 2000; Zlatković et al, 2010 |
| **L6** | Prvonek | 42°30' N / 22°05' E  South Serbia | 580 | 65 | Banjska river | Water supply | Artificial | Dević et al., 2014 |
| **L7** | Ćelije | 43°24' N / 21°10' E  South Serbia | 284 | 41 | Rasina river | Water supply, irrigation, flood defense | Artificial | Milenković Andjelković et al., 2010; Stanković, 2000 |
| **L8** | Vrutci | 43°50' N / 19°42' E  West Serbia | 621 | 50 | Đetinja river | Water supply | Artificial | Kostić et al., 2016 |
| **L9** | Garaši | 44°17' N / 20°29' E  Central Serbia | 268 | 26 | Bukulja river | Water supply, Fishing | Artificial | Dević et al., 2014; Stanković, 2000 |
| **L10** | Grlište | 43°49' N / 22°13' E  East Serbia | 193  159 HMZ | 20 | Grliška river | Water supply, sports fishing, recreation | Artificial | Dević et al., 2014 |

^1^ - m a.s.l. – meters above sea level

^2^ Intended uses according to Anonymous (2001).

**Table S2.** ICP-OES: Limit of detection (LOD) and Limit of quantification (LOQ)

| **Element** | **LOD**  (µg/L) | **LOQ**  (µg/L) |
| --- | --- | --- |
| Ca | 0.12 | 0.69 |
| Mg | 0.14 | 0.94 |
| K | 0.22 | 1.21 |
| Na | 0.034 | 0.126 |
| Si | 0.38 | 1.25 |
| As | 0.013 | 0.045 |
| Al | 0.076 | 0.381 |
| B | 0.78 | 3.12 |
| Ba | 0.012 | 0.054 |
| Cd | 0.028 | 0.091 |
| Co | 0.25 | 0.95 |
| Cr | 0.081 | 0.029 |
| Cu | 0.19 | 0.67 |
| Fe | 0.16 | 0.54 |
| Li | 0.056 | 0.190 |
| Mn | 0.034 | 0.115 |
| Ni | 0.15 | 0.59 |
| Pb | 0.21 | 0.78 |
| Sb | 0.24 | 0.87 |
| Se | 0.12 | 0.45 |
| Sr | 0.016 | 0.062 |
| V | 0.18 | 0.74 |
| Zn | 0.057 | 0.180 |

**Table S3.** Concentrations of macro elements in water of studied lakes (mg/L)

| **Element** | **Ca** | **Mg** | **K** | **Na** | **Si** |
| --- | --- | --- | --- | --- | --- |
| **Lake** |  |  | mg/L |  |  |
| Sava (L1) | 30.16^c^±0.28^1^ | 12.58^e^±0.37 | 1.43^de^ ±0.06 | 7.86^c^±0.16 | 0.08^g^±0.01 |
| Gruža (L2) | 31.65^bc^±0.43 | 15.09^d^±0.43 | 3.41^a^ ±0.08 | 8.41^b^±0.16 | 1.78^e^ ±0.07 |
| Srebrno (L3) | 32.54^b^±0.53 | 25.54^b^±0.53 | 3.48^a^±0.14 | 15.48^a^±0.23 | 4.36^c^±0.14 |
| Vlasina (L4) | 11.50^g^±0.60 | 2.42^j^±0.09 | 0.62^f^±0.03 | 2.45^g^±0.12 | 2.08^d^ ±0.07 |
| Bovan (L5) | 50.03^a^±0.81 | 10.57^f^±0.16 | 2.22^b^±0.07 | 7.49^c^±0.12 | 1.25^f^ ±0.06 |
| Prvonek (L6) | 20.47^e^±0.36 | 5.62^h^±0.21 | 1.46^d^±0.04 | 3.98^f^±0.10 | 6.09^a^ ±0.11 |
| Ćelije (L7) | 23.51^d^±0.48 | 21.95^c^±0.90 | 1.92^c^±0.10 | 5.68^d^±0.13 | 0.12^g^ ±0.01 |
| Vrutci (L8) | 30.66^bc^±0.62 | 27.25^a^±0.70 | 0.60^f^±0.02 | 2.07^g^±0.08 | 4.79^b^±0.13 |
| Garaši (L9) | 15.49^f^±0.38 | 4.49^h^±0.22 | 1.51^d^±0.07 | 5.57^d^±0.16 | 1.72^e^±0.06 |
| Grlište (L10) | 50.69^a^±1.39 | 9.09^g^±0.31 | 1.23^e^±0.06 | 4.91^e^±0.14 | 0.29^g^ ±0.02 |
| Max | 50.69 | 27.25 | 3.48 | 15.48 | 6.09 |
| Min | 11.50 | 2.42 | 0.60 | 2.07 | 0.08 |

^1^ Results are presented as mean ± standard deviation

Different letters (a,b,c…) within the column present statistically significant differences between samples (p<0.05)

**Table S4.** Concentrations of micro- and trace elements in water of studied lakes (μg/L)

| **Element** | **As** | **Al** | **B** | **Ba** | **Cd** | **Co** | **Cr** | **Cu** | **Fe** |
| --- | --- | --- | --- | --- | --- | --- | --- | --- | --- |
| **Lake** |  |  |  |  | μg/L |  |  |  |  |
| Sava (L1) | 8.05^b^±0.10**^1^** | 225.90^b^±5.40 | 23.23^b^±0.05 | 20.11^d^±0.34 | /**^2^** | 0.08^e^±0.01 | / | 3.50^b^±0.09 | 10.27^j^±0.26 |
| Gruža (L2) | 7.30^c^±0.17 | 41.07^d^±1.06 | 20.83^c^±0.95 | 44.16^b^±0.91 | / | 0.19^d^±0.01 | 0.15^cd^±0.01 | 3.87^a^±0.12 | 73.39^c^±0.51 |
| Srebrno (L3) | 9.47^a^±0.39 | / | 35.97^a^±0.65 | 28.54^c^ ±0.53 | / | 0.20^d^±0.01 | 0.18^c^±0.01 | 2.19^c^±0.07 | 19.37^g^±0.34 |
| Vlasina (L4) | 2.70^g^ ±0.08 | 16.42^e^±0.20 | 0.44^f^±0.02 | 2.55^h^±0.11 | / | 0.18^d^ ±0.01 | 0.09^e^±0.02 | 0.96^f^±0.04 | 40.83^e^±0.74 |
| Bovan (L5) | 5.18^d^±0.25 | 0.83^f^±0.01 | 17.70^d^±0.60 | 56.17^a^±0.36 | / | 0.04^f^±0.01 | 0.02^f^±0.01 | 1.84^d^±0.07 | 14.00^h^±0.50 |
| Prvonek (L6) | 3.89^f^±0.18 | 74.97^c^±0.06 | 0.75^f^±0.01 | 28.22^c^±0.23 | / | 0.26^bc^±0.01 | 0.12^de^±0.01 | 1.62^e^±0.07 | 92.57^b^±0.76 |
| Ćelije (L7) | 5.10^d^±0.20 | 19.56^e^±0.23 | 20.27^c^±0.35 | 19.42^d^±0.23 | / | 0.25^c^±0.01 | 0.63^b^±0.01 | 1.69^de^±0.08 | 36.02^f^±0.90 |
| Vrutci (L8) | 2.17^g^±0.06 | 35.14^d^±0.37 | 24.50^b^±0.50 | 4.98^g^±0.21 | / | 0.28^b^±0.01 | 1.43^a^±0.04 | 1.84^d^±0.06 | 54.77^d^±0.76 |
| Garaši (L9) | 4.53^e^±0.20 | 269.07^a^±2.55 | 5.33^e^±0.15 | 10.89^f^±0.55 | / | 0.33^a^±0.00 | / | 1.62^e^±0.08 | 191.30^a^±1.40 |
| Grlište (L10) | 0.35^h^±0.02 | / | 3.51^e^±0.10 | 16.17^e^±0.95 | 0.04 | / | / | / | 2.25^m^±0.13 |
| Max | 9.47 | 269.07 | 35.97 | 56.17 |  | 0.33 | 0.02 | 3.87 | 191.30 |
| Min | 0.35 | 0.83 | 0.44 | 2.55 |  | 0.04 | 1.43 | 0.96 | 2.25 |

**^1^** Results are presented as mean ± standard deviation; Different letters (a,b,c…) within the column present statistically significant differences between samples (p<0.05)

**^2^** / Below the LOD

**Table S4.** Continued…

| **Element** | **Li** | **Mn** | **Ni** | **Pb** | **Sb** | **Se** | **Sr** | **V** |
| --- | --- | --- | --- | --- | --- | --- | --- | --- |
| **Lake** |  |  |  |  | μg/L |  |  |  |
| Sava (L1) | 2.64^f^±0.13 | 0.98^g^±0.01 | 0.15^efg^±0.01 | 0.88^cd^±0.03 | 1.12^c^±0.04 | 0.17^e^±0.01 | 105.80^e^±2.70 | 0.86^d^±0.02 |
| Gruža (L2) | 3.41^e^±0.08 | 19.18^d^±0.17 | 1.37^c^±0.07 | 1.62^a^±0.07 | 1.11^c^±0.03 | 0.18^e^±0.01 | 161.70^c^±2.90 | 2.65^a^±0.04 |
| Srebrno (L3) | 5.17^b^±0.09 | 69.30^b^±0.19 | 0.32^e^±0.01 | 0.77^de^±0.04 | 0.44^f^±0.02 | 0.65^c^±0.02 | 180.60^b^±2.70 | 0.59^f^±0.02 |
| Vlasina (L4) | 0.81^h^±0.04 | 17.56^d^±0.34 | / | 1.39^b^±0.06 | 0.96^d^±0.03 | 0.73^b^±0.02 | 39.38^g^±0.49 | 0.27^g^±0.01 |
| Bovan (L5) | 3.63^de^±0.04 | 9.51^e^±0.11 | 0.20^ef^±0.01 | 0.99^c^±0.04 | 1.87^a^±0.09 | 0.15^e^±0.01 | 181.60^b^±2.70 | 0.56^f^±0.01 |
| Prvonek (L6) | 1.42^g^±0.07 | 254.80^a^±1.20 | 0.01^fg^±0.001 | 0.80^de^±0.04 | 1.05^cd^±0.04 | 0.74^b^±0.03 | 116.40^de^±2.20 | 0.72^e^±0.01 |
| Ćelije (L7) | 3.85^d^±0.08 | 11.33^e^±0.24 | 2.04^b^±0.05 | 0.68^e^±0.03 | 0.59^e^±0.03 | 0.32^d^±0.01 | 118.30^d^±2.00 | 1.39^c^±0.07 |
| Vrutci (L8) | 4.66^c^±0.11 | 4.05^f^±0.07 | 9.96^a^±0.19 | 0.76^de^±0.03 | 1.33^b^±0.06 | 1.23^a^±0.04 | 57.44^f^±0.49 | 1.63^b^±0.06 |
| Garaši (L9) | 10.56^a^±0.15 | 47.36^c^±0.45 | 0.53^d^±0.02 | 1.33^b^±0.06 | 0.37^f^±0.01 | / | 61.53^f^±0.68 | 0.70^e^±0.02 |
| Grlište (L10) | 0.98^h^±0.04 | 2.05^fg^±0.06 | / | / | / | / | 225.30^a^±11.10 | 1.48^c^±0.05 |
| Max | 10.56 | 254.80 | 9.96 | 1.62 | 1.87 | 1.23 | 225.30 | 2.65 |
| Min | 0.81 | 0.98 | 0.01 | 0.68 | 0.37 | 0.15 | 39.38 | 0.27 |

^1^ Results are presented as mean ± standard deviation; Different letters (a,b,c…) within the column present statistically significant differences between samples (p<0.05)

^2^ / below the LOD

**Table S5**. Limit values of national and international regulations and range of elements concentrations in the lake water samples L1-L10

|  | **Regulation on limit values of pollutants in surface and ground waters and sediments and deadlines for their achievement**  (Official Gazette of RS, 2014) | | | | | | | | | **National Primary Drinking Water Regulations** (EPA, 2009) | **Guidelines for drinking-water quality**  (WHO, 2006) |  | Range of elements concentrations and pH in lake water samples  L1-L10 |
| --- | --- | --- | --- | --- | --- | --- | --- | --- | --- | --- | --- | --- | --- |
|  | **Class I** | | **Class II** | | **Class III** | **Class IV** | | **Class V** | |  |  |  |  |
|  |  | |  | | **LV^1^** (μg/L) |  | |  | | **MCL^2^/SMCL^3^** (mg/L) | **RV^4^** (mg/L) |  | mg/L |
|  |  | |  | |  |  | |  | |  | 75 | **Ca** | 11.50 – 50.69 |
|  |  | |  | |  |  | |  | |  | 30 | **Mg** | 2.42 – 27.25 |
|  |  | |  | |  |  | |  | |  | 12 | **K** | 0.60 – 3.48 |
|  |  | |  | |  |  | |  | |  | 50 | **Na** | 2.07 – 15.48 |
|  |  | |  | |  |  | |  | |  |  |  | μg/L |
| **Al** |  | |  | |  |  | |  | | 0.05-2**^3^** |  | **Al** | 0.83 – 269.07 |
| **As** | <5 (or Nl^*^) | | 10 | | 50 | 100 | | >100 | | 0.01**^2^** |  | **As** | 2.17 – 9.47 |
| **B** | 300 (or Nl) | | 1000 | | 1000 | 2500 | | >2500 | |  |  |  | 0.44 – 35.97 |
| **Ba** | / | | / | | / | / | | / | | 2**^2^** |  | **Ba** | 2.55 – 56.17 |
| **Cd** | / | | / | | / | / | | / | | 0.005**^2^** |  | **Cd** | 0.04 |
| **Cr** | 25 (or Nl) | | 50 | | 100 | 250 | | >250 | | 0.1**^2^** _(total Cr)_ |  | **Cr** _(total)_ | 0.02 – 1.43 |
| **Cu** | 5 /**^**^**H=10  22/H=50  40/H=100  112/H=300 | | 5 /H=10  22/H=50  40/H=100  112/H=300 | | 500 | 1000 | | >1000 | | 1.3 _(Action Level)_  1**^63^** |  | **Cu** | 0.96 – 3.87 |
| **Fe** | 200 | | 500 | | 1000 | 2000 | | >2000 | | 0.3**^3^** |  | **Fe** | 2.25 – 191.30 |
| **Hg** | / | | / | | / | / | | / | | 0.002**^2^** |  | **Hg** _inorganic)_ |  |
| **Mn** | 50 | | 100 | | 300 | 1000 | | >1000 | | 0.05**^3^** | 0.1 | **Mn** | 0.98 – 254.80 |
| **Ni** | / | | / | | / | / | | / | |  |  |  | 0.01 – 9.96 |
| **Pb** | / | | / | | / | / | | / | | 0.015 _(Action Level)_ |  | **Pb** | 0.68 – 1.62 |
| **Sb** | / | | / | | / | / | | / | | 0.006 |  | **Sb** |  |
| **Zn** | 30 /H=10  200/H=50  300/H=100  300/H=500 | | 300 /H=10  700/H=50  1000/H=100  2000/H=500 | | 2000 | 5000 | | >5000 | | 5**^3^** |  | **Zn** | <LOQ**^5^** |
| **pH** | 6.5–8.5 | 6.5–8.5 | | 6.5–8.5 | | | 6.5–8.5 | | <6.5 / >8.5 | 6.5–8.5 | 6.5–8.5 |  | 7.45-8.67 |

**^1^LV** - Limit values for pollutants in surface water (Official Gazette of RS, 24/2014)

/– Not regulated; ^*^Nl – Natural level; ^**^H – Hardness (mg/L CaCO_3_)

According to the national regulations surface waters are classified in five classes (class I>class II>class III>class IV>class V), Table S5. Waters classified within I–IV classes may be used for drinking water supply (after the treatment by appropriate methods for water purification), bathing and recreation, irrigation, industrial use (process and cooling water), whereas water ranked as class V cannot be used for any purpose (Official Gazette of RS, 2014)

**^2^MCL** - Maximum Contaminant Levels in drinking water (National Primary Drinking Water Regulations, EPA 816-F-09-004, 2009)

**^3^ SMCL**- Secondary Maximum Contaminant Level (National Primary Drinking Water Regulations, EPA 816-F-09-004, 2009)

**^4^ RV-** recommended values of water quality parameters for drinking water (WHO, 2006)

**^5^** < LOQ – below the limit of quantification

**References**

Anonymous (2001). Water management base of the Republic of Serbia, Institute for Water Management "Jaroslav Černi" Belgrade, Ministry of Agriculture, Forestry and Water Management, <http://www.srbijavode.rs/Data/Files/vodoprivredna_osnova_republike_srbije.pdf> (*in Serbian*)

Dević, G., Đorđević, D., & Sakan, S. (2014). Freshwater environmental quality parameters of man-made lakes of Serbia. *Environmental Monitoring and Assessment*, 186, 5221–5234. DOI 10.1007/s10661-014-3771-0

Kostić, D., Marjanović, P., Marjanović, M., Blagojević, A., Trbojević, I., Predojević, D., Subakov Simić, G., Vulić, D., Obradović, V., & Naunović, Z. (2016). Drivers of phytoplankton blooms in the Vrutci reservoir during 2014-2015 and implications for water supply and management, *Water Research and Management*, 6, 3-12.

Martinović-Vitanović, V., Popović, N., Ostojić, S., Raković, M., Kalafatić, V. (2009). First investigations of the water quality of the lake Srebrno in Serbia based on saprobiological analysis of benthic fauna. *Romanian Journal of Biology – Zoolog*y, 54, 151-165.

Mićković, B., Nikčević, M., Grozdić, T., Pucar, M., Hegedi, A., & Gačić, Z. (2014). Ecological potential assessment of Sava Lake based on fish community composition: preliminary results, *Water Research and Management*, 4, 21-25.

Milenković Andjelković, A., Nikolić, D., & Anđelković, M. (2010). Investigation ecological condition and water quality of Lake Ćelije. *EUROINVENT*, *1*, 88-93

National Primary Drinking Water Regulations, EPA 816-F-09-004. (2009). https://www.epa.gov/sites/default/files/2016-06/documents/npwdr_complete_table.pdf (Accessed on 20 June 2022)

Regulation on the limit values of priorities i priority hazardous substances that pollute surface waters and deadlines for their achievement". (2014). Official Gazette of RS, 24/2014. <https://www.ecolex.org/details/legislation/regulation-on-limit-values-of-priority-and-priority-hazardous-substances-that-pollute-the-surface-waters-and-deadlines-for-their-achievement-lex-faoc155342/>?

Stanković, S. (2000). *Jezera Srbije – limnološka monografija*. Srpsko Geografsko Društvo (SGD), Beograd. (*in Serbian*)

Zlatković, S.; Šabić, D.; Milinčić, M.; Knežević-Vukčević, J.; Stanković, S. (2010). Geographical and biological analysis of the water quality of Bovan lake, Serbia, *Archives of Biological Sciences,* 62, 1083-1087. DOI: 10.2298/ABS1004083Z

WHO. (2006). Guidelines for drinking-water quality, volume 1—recommendations (3rd ed.). Geneva: Word Health Organization
